# IL-33 Promotes CD11b/CD18-Mediated Adhesion of Eosinophils to Cancer Cells and Synapse-Polarized Degranulation Leading to Tumor Cell Killing

**DOI:** 10.3390/cancers11111664

**Published:** 2019-10-26

**Authors:** Sara Andreone, Francesca Spadaro, Carla Buccione, Jacopo Mancini, Antonella Tinari, Paola Sestili, Adriana Rosa Gambardella, Valeria Lucarini, Giovanna Ziccheddu, Isabella Parolini, Cristiana Zanetti, Maria Teresa D’Urso, Adele De Ninno, Luca Businaro, Claudia Afferni, Fabrizio Mattei, Giovanna Schiavoni

**Affiliations:** 1 Department of Oncology and Molecular Medicine, Istituto Superiore di Sanità, 00161 Rome, Italy; sara.andreone93@hotmail.it (S.A.); carla.buccione@gmail.com (C.B.); jacopo.mancini27@gmail.com (J.M.); adriana.gambardella@libero.it (A.R.G.); valeria.lucarini@opbg.net (V.L.); giovanna.ziccheddu@gmail.com (G.Z.); isabella.parolini@iss.it (I.P.); cristiana.zanetti@iss.it (C.Z.); mariateresa.durso@iss.it (M.T.D.); fabrizio.mattei@iss.it (F.M.); 2 Microscopy Unit, Core Facilities, Istituto Superiore di Sanità, 00161 Rome, Italy; francesca.spadaro@iss.it (F.S.); paola.sestili@iss.it (P.S.); 3 Center for Gender Medicine, Istituto Superiore di Sanità, 00161, Rome, Italy; antonella.tinari@iss.it; 4 Institute for Photonics and Nanotechnologies, National Research Council (CNR), 00156 Rome, Italy; adeledeninno@gmail.com (A.D.N.); luca.businaro@cnr.it (L.B.); 5 National Center for Drug Research and Evaluation, Istituto Superiore di Sanità, 00161 Rome, Italy; claudia.afferni@iss.it

**Keywords:** interleukin (IL)-33, eosinophils, degranulation, adhesion, tumor cell killing, mouse tumor models

## Abstract

Eosinophils are major effectors of Th2-related pathologies, frequently found infiltrating several human cancers. We recently showed that eosinophils play an essential role in anti-tumor responses mediated by immunotherapy with the ‘alarmin’ intereukin-33 (IL-33) in melanoma mouse models. Here, we analyzed the mechanisms by which IL-33 mediates tumor infiltration and antitumor activities of eosinophils. We show that IL-33 recruits eosinophils indirectly, via stimulation of tumor cell-derived chemokines, while it activates eosinophils directly, up-regulating CD69, the adhesion molecules ICAM-1 and CD11b/CD18, and the degranulation marker CD63. In co-culture experiments with four different tumor cell lines, IL-33-activated eosinophils established large numbers of stable cell conjugates with target tumor cells, with the polarization of eosinophil effector proteins (ECP, EPX, and granzyme-B) and CD11b/CD18 to immune synapses, resulting in efficient contact-dependent degranulation and tumor cell killing. In tumor-bearing mice, IL-33 induced substantial accumulation of degranulating eosinophils within tumor necrotic areas, indicating cytotoxic activity in vivo. Blocking of CD11b/CD18 signaling significantly reduced IL-33-activated eosinophils’ binding and subsequent killing of tumor cells, indicating a crucial role for this integrin in triggering degranulation. Our findings provide novel mechanistic insights for eosinophil-mediated anti-tumoral function driven by IL-33. Treatments enabling tumor infiltration and proper activation of eosinophils may improve therapeutic response in cancer patients.

## 1. Introduction

Eosinophils are a subset of granulocytes mostly known for their capacity to stimulate allergic reactions and to fight parasites. Under normal conditions, these cells are relatively rare representing 1–3% of total white blood cells. During certain inflammatory conditions (i.e., allergies, parasitic infections, autoimmune diseases) eosinophils can rapidly increase, due to both de novo generation from bone marrow precursors and proliferation of tissue-resident cells, infiltrating inflamed tissues, including tumors [1]. Tumor-associated tissue eosinophilia has been described in various solid tumors although its prognostic value in clinical oncology remains controversial [2]. In vivo, eosinophils were shown to exert a protective role inhibiting tumor growth in mouse models of melanoma [3,4,5,6], colorectal cancer [7], hepatocellular carcinoma [8], and inducible fibrosarcoma [9]. Furthermore, eosinophils infiltration in the lung can prevent the formation of experimental pulmonary metastasis [3,4,6].

Eosinophils are equipped with granules carrying a plethora of cytotoxic mediators, such as the cationic proteins major basic protein (MBP), eosinophil cationic protein (ECP), eosinophil peroxidase (EPX), and granzymes (granzyme-A in human cells, granzyme-B in murine cells) that can destroy target cells or tissues [10]. Indeed, eosinophils were shown to directly kill human colon carcinoma cells via release of granzyme-A in a mechanism dependent on the integrin CD11a/CD18 and on the cytokine IL-18 [11,12]. In addition, eosinophils activated by cross-linking of the receptor 2B4/CD244 exhibited tumoricidal activities against human B lymphoma cells [13]. It appears that the process of adhesion through integrins is important for eosinophil activation during inflammation [14]. However, it is unclear whether cell adhesion and degranulation are sequential or independent events. In addition, the mechanisms of granule protein secretion (i.e., degranulation) in eosinophils leading to tumor cytotoxicity remain to be defined.

IL-33 is an epithelial-derived “alarmin” playing multiple functions in Th2-related immunopathologies and recently implied in cancer immunity, exerting pro- or anti-tumoral activities, depending on the tumor type and microenvironmental factors [15]. Tumor over-expression or exogenous administration of IL-33 promotes anti-tumor immune responses in vivo in models of melanoma [6,16,17,18,19], acute myeloid leukemia [20], lung [16,21] and colorectal cancer [22]. IL-33 drives substantial recruitment of eosinophils to inflamed sites, such as the lung, likely by promoting eosinophilopoiesis in the bone marrow [23,24]. In addition, IL-33 can induce eosinophil superoxide anion production [25] and promote the survival of eosinophils [26]. We initially reported that IL-33 inhibits tumor growth and pulmonary metastasis in vivo in an eosinophil-dependent manner [6]. Subsequent studies confirmed eosinophil-mediated anti-tumoral effects of IL-33 in pre-clinical models of metastatic peritoneal cancer [27], hepatocellular, and prostatic cancer [19]. Our study suggested that eosinophils recruited by IL-33 might either function as accessory cells attracting CD8 T cells or play a direct antitumor role. Here, we further investigated the anti-tumoral mechanisms operated by eosinophils following activation with IL-33. By using a panel of tumor cell lines, we show that IL-33 promotes tumoricidal functions of eosinophils in vitro and in vivo in a cell adhesion-dependent manner, through the integrin CD11b/CD18 and by inducing lytic granule convergence to the immune synapse. Our data provide mechanistic insights into eosinophil anti-tumor activities stimulated by IL-33 that involve cell contact and directional secretion of granules toward target tumor cells enhancing killing efficiency.

## 2. Results

### 2.1. IL-33 Recruits Eosinophils to the Tumor Site via Indirect Mechanism

**Figure 1:**
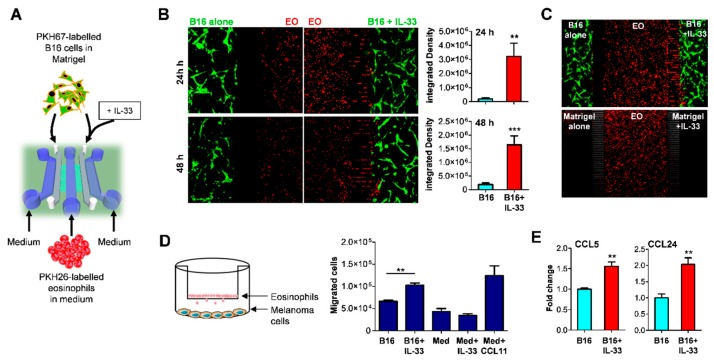
IL-33 recruits eosinophils to the tumor site via indirect mechanisms. (**A**) Schematic view of microfluidic chip employed to monitor the migration of eosinophils towards tumor cells and in response to IL-33 in a competitive setting. B16.F10 melanoma cells were labeled with PKH67 dye (green), embedded in Matrigel with or without the addition of IL-33 and inserted in lateral gel chambers (grey). Eosinophils (EO) were labelled with PKH26 dye (red), resuspended in medium and inserted in the central chamber (blue). The lateral chambers (blue) were filled with medium. Chips were placed in a humidified CO_2_ incubator. The migration of EO towards lateral tumor-gel chambers was monitored by fluorescence microscopy. (**B**) Preferential migration of EO (red) towards IL-33-treated B16 tumor cells (green) after 24 and 48 h culture (left) and relative quantitative analysis (right), expressed by integrated density of red fluorescence in the two Matrigel chambers. Mean of several fields ±SD, from 3 replicates. (**C**) EO migrate towards IL-33-treated B16 cells but do not in response to IL-33 alone in a chip. (**D**) Transwell migration assay of EO in response to IL-33 or B16 cells treated with IL-33. CCL11 was used as a positive control. Med, medium. Data show the number of eosinophils migrated to the lower compartment in culture triplicates ±SD. One representative experiment out of two is shown. (**E**) Induction of EO-attracting chemokines CCL5 and CCL4 by IL-33 in B16 melanoma cells, as revealed by qRT-PCR. ** *p* < 0.01.

Our previous studies indicated that injection of IL-33 in mice bearing B16.F10 melanoma tumors determined massive infiltration of eosinophils in vivo [6]. This finding suggested that either IL-33 attracted eosinophils directly, as seen with other immune cells [28] or indirectly, via stimulation of tumor-released chemokines. To address this issue, we employed an organ-on-chip approach, a microfluidic-based technology recently developed in our laboratories as a reliable tool to measure tumor-immune cell interactions [29,30]. An ad hoc fabricated device, composed of three main fluidic chambers and two narrow gel-containing chambers, interconnected by two arrays of microchannels, was used to co-culture bone marrow-derived eosinophils (EO) with melanoma cells (Figure 1A). In this device for competitive assay [31], PKH67 green-labeled B16 melanoma cells are loaded in the two opposite gel chambers alone or with added IL-33 and confronted for their capacity to attract PKH26 red-labeled EO, loaded into the middle fluidic chamber (Figure 1A). As denoted by red fluorescence distribution, EO exhibited notable displacement towards the chambers containing IL-33-treated melanoma cells after 24 h, occupying the right-side microchannels and tumor-Matrigel chambers (Figure 1B). Of note, when the device contained Matrigel alone (without melanoma cells), addition of IL-33 failed to elicit the migration of EO (Figure 1C), indicating that IL-33 triggered a chemotactic response via stimulation of tumor cells. These observations were confirmed in a Transwell migration assay where eosinophils were tested for their chemotactic migration towards IL-33 or IL-33 treated melanoma cells. Here, addition of IL-33 to the lower compartment induced the migration of EO from the upper chamber only in presence of melanoma cells in the bottom chamber (Figure 1D). We next assessed whether IL-33 induced the expression of eosinophil-attracting chemokines (CCL11, CCL24, CCL26, and CCL5) [32] in melanoma cells and found significant up-regulation of CCL5 and CCL24 (Figure 1E). Neither CCL11 nor CCL26 were expressed by melanoma cells (data not shown). Thus, IL-33 recruits tumor-infiltrating eosinophils indirectly, via stimulation of chemokine production at least in part by tumor cells.

### 2.2. IL-33 Activates Eosinophils Directly and Promotes Tumor Cell Killing

**Figure 2:**
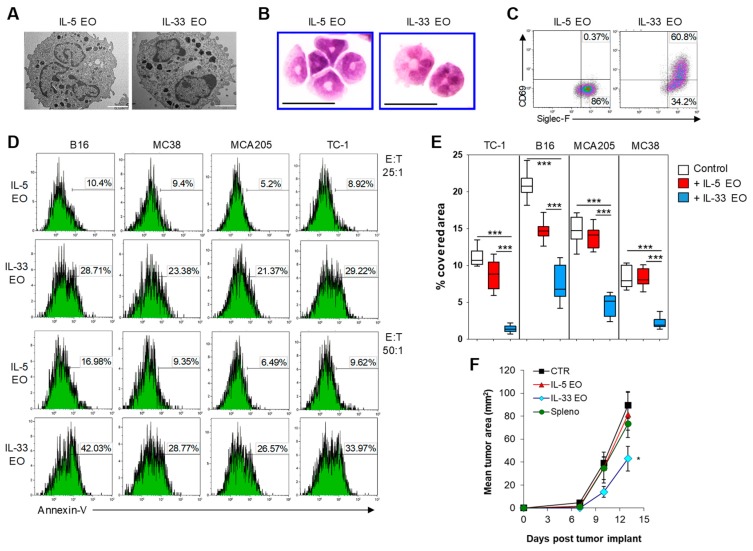
IL-33 directly activates eosinophils and promotes tumoricidal activity. (**A**) Representative electron micrographs of bone marrow-derived eosinophils classically differentiated with IL-5 (IL-5 EO) or terminally activated with IL-33 (IL-33 EO). In both eosinophils, typical granules containing electron-dense crystals were visible. (**B**) Morphology of IL-5 or IL-33-activated eosinophils. Cytospins were prepared and eosinophils were stained with hematoxylin/eosin. Bars represent 10 μm. (**C**) Flow cytometry analysis of CD69 and Siglec-F expression in IL-33 EO and IL-5 EO. (**D**) Induction of tumor apoptosis by IL-5 vs. IL-33 EO on the indicated tumor cell lines after co-culture at the indicated E:T ratios. (**E**) IL-5 EO and IL-33 EO were co-cultured with TC-1, B16, MCA205 or MC38 tumor cells then washed out and adherent tumor cells were stained with Crystal Violet. Quantitative analysis of the tumor-covered area in the indicated culturing conditions is shown. Data are represented as fraction of area occupied by the indicated tumor cells with respect to the total field area. Mean values ±SD from 10 different microphotographs per condition are shown. *** *p* < 0.001. (**F**) EO tumoricidal activity in vivo. IL-5 EO or IL-33 EO were co-injected with B16.F10 melanoma cells subcutaneously into syngeneic C57Bl/6 mice. Control groups consisted of mice receiving B16 cells alone (CTR) or B16 cells plus splenocytes from naïve mice (Spleno). Tumor growth was measured. Mean tumor area ±SEM of 10 mice from 2 independent experiments is shown. * *p* < 0.05.

We recently reported that the terminal differentiation of BM-derived EO with IL-33, obtained by culturing BM cells in presence of IL-5 for the first 10 days of culture followed by IL-33 for the last 6 days of culture, results in the generation of highly activated EO [6]. We further characterized these IL-33-activated EO (IL-33 EO) compared with eosinophils differentiated with IL-5 for the whole culturing time (IL-5 EO). Transmission electron microscopy (TEM) showed similar ultrastructural organization in both eosinophil preparations, with presence of electron-dense granules (Figure 2A). However, cytospin preparations revealed that while IL-5 EO retained round shaped nuclei, IL-33 EO exhibited pluri-lobated nuclei indicative of a more mature phenotype (Figure 2B). Furthermore, IL-33 EO expressed higher levels of the activation marker CD69 compared to IL-5 EO (Figure 2C). We previously reported that IL-33-activated EO were superior at killing target melanoma cells [6]. To extend these findings, we tested the tumoricidal activity of IL-33 EO against four different tumor cell lines (B16, MC38, MCA205, and TC-1). 

Tumor cells were labeled with PKH26 red fluorescent dye and cultured in the presence or absence of eosinophils at different E:T ratios for 5 h. Tumor cell death was measured by Annexin V staining in PKH26^+^ tumor cells. As shown in Figure 2D, IL-33 EO induced rapid apoptosis of tumor cells with greater efficiency than IL-5 EO. Tumor cell death was further confirmed by a significant reduction of tumor-covered area after 24 h co-culture with IL-33 EO (Figure 2E, Figure S1). Furthermore, IL-33 EO, unlike IL-5 EO, interfered with tumor 3D-spheroid formation in vitro (Figure S2) and slowed tumor outgrowth in vivo when co-injected with B16 melanoma cells into syngeneic mice (Figure 2F). Together, these findings indicate that IL-33 greatly enhances the tumoricidal properties of eosinophils resulting in tumor growth suppression in vivo.

### 2.3. IL-33 Promotes Adhesion of Eosinophils to Tumor Cells and Subsequent Lytic Granule Convergence

**Figure 3:**
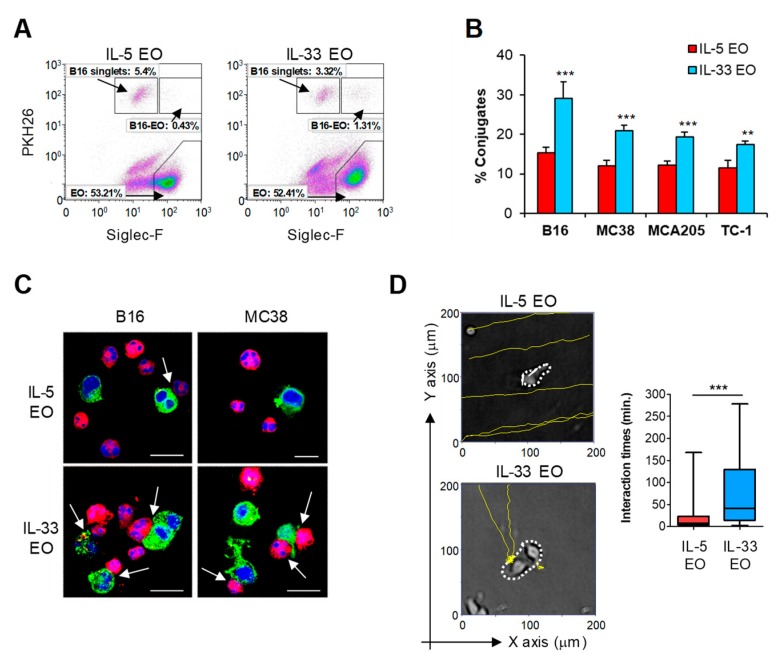
Increased tumor cell adhesion in IL-33 activated eosinophils. (**A**) Analysis of cell conjugate formation among Siglec-F^+^ IL-5 EO and IL-33 EO and PKH26-labeled B16 melanoma cells. (**B**) Formation of cell conjugates among IL-5 EO and IL-33 EO and the indicated tumor cell lines. Mean ±SD of three experiments. (**C**) CLSM analysis of cell conjugates among PKH26-labeled IL-5 or IL-33 EO and PKH67-labeled B16 or MC38 tumor cells. Arrows indicate cell conjugates among eosinophils (red) and tumor cells (green). Scale bars, 20 µm. Images are representative of 3 independent experiments. (**D**) Analysis of interactions between eosinophils and tumor cells. Tracking analysis of time lapse video recordings was performed by the TrackMate plugin of ImageJ software. Yellow lines represent trajectories of eosinophils. White dotted lines depict the target tumor cell. One experiment out of three is shown. Histograms represent mean interaction times obtained from several regions (±SD). *** *p* < 0.001.

Cell adhesion is one important mechanism accounting for the effector functions of eosinophils in several pathologies, such as asthma [33]. Therefore, we conducted a cell-cell adhesion assay to evaluate whether the increased tumoricidal function of IL-33-activated EO could be ascribed to enhanced cell contact. In this assay, PKH26-labeled tumor cells were co-cultured with eosinophils for 90 min and then analyzed for the formation of cell-cell conjugates by flow cytometry [11,12]. Analysis of Siglec-F^+^PKH26^+^ cells over the total of PKH26^+^ tumor cells revealed that IL-33 EO formed increased numbers of cell conjugates with tumor cells, as compared to IL-5 EO (Figure 3A,B). CLSM analysis confirmed the effective establishment of cell conjugates among PKH26^+^ eosinophils and PKH67^+^ tumor cells, with notably increased incidence in IL-33 EO (Figure 3C). Furthermore, time-lapse video microscopy revealed that IL-33-activated EO had higher propensity to migrate towards and interact with tumor cells, in comparison with IL-5 EO (Figure 3D, Figure S3, Videos S1–S4). 

**Figure 4:**
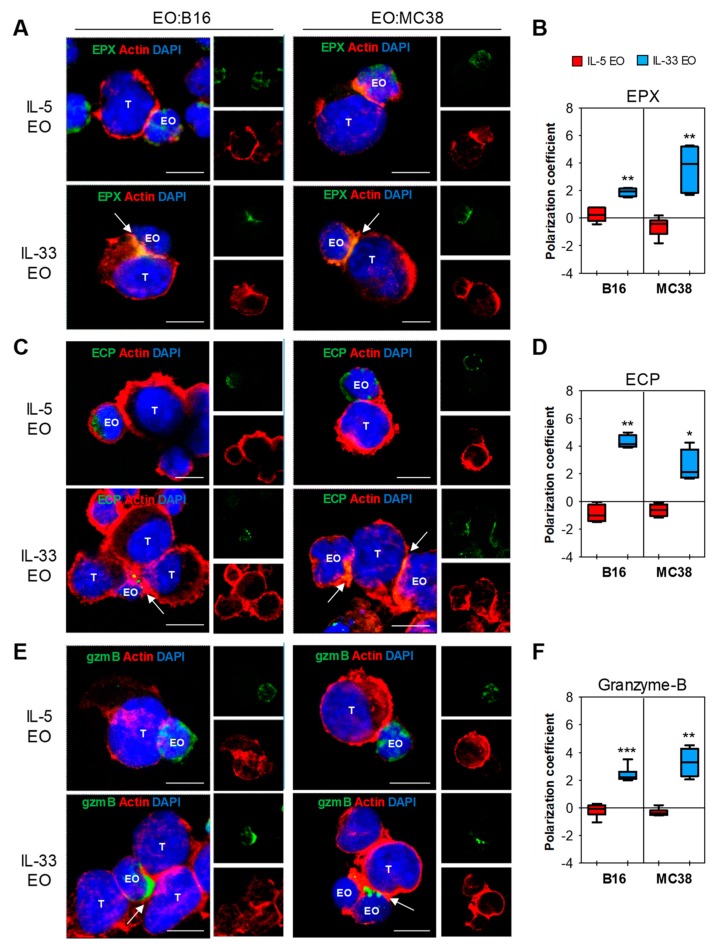
Polarization of granule proteins at immune synapses in IL-33 activated eosinophils. (**A**,**C**,**E**) CLSM observations of EO-tumor cell conjugates (central optical sections). IL-5 EO or IL-33 EO were allowed to conjugate with B16 (left panels) or MC38 (right panels) tumor cells. Cell conjugates stained for EPX (**A**), ECP (**C**) or granzyme-B (GzmB) (**E**) expression, all detected in green. Actin staining was performed with Alexa Fluor^®^-594 phalloidin (red). Nuclei are reported in blue (DAPI). Co-localization of granule-derived proteins and actin at immune synapses of IL-33 EO is shown in merged images, detected in yellow and indicated by arrows. Inserts represent separate channel images. Scale bars, 10 µm. Representative examples of 5 independent experiments are shown. (**B**,**D**,**F**) Quantitative determination of protein polarization to the immune synapses by ImageJ software. Data are expressed as the mean polarization coefficient of several images depicting cell conjugates from 5 independent experiments ±SD. * *p* < 0.05, ** *p* < 0.01, *** *p* < 0.001.

Next, we analyzed the expression of the granule cationic proteins EPX and ECP, and of granzyme-B. CLSM showed higher expression of granzyme-B in IL-33 EO, compared with IL-5 EO (Figure S4), confirming that IL-33 can increase this protein in eosinophils [6]. In contrast, EPX and ECP were expressed at similar levels in both eosinophil types (Figure S4). Of note, when eosinophils were co-cultured with tumor cells, a marked polarization of EPX (Figure 4A,B), ECP (Figure 4C,D), and granzyme-B (Figure 4E,F) to the immune synapses was observed selectively in IL-33 EO. In contrast, in co-cultures with IL-5 EO these molecules appeared uniformly distributed within the intracellular compartments of eosinophils (Figure 4). Taken together, these data demonstrate that IL-33 stimulates both adhesions of eosinophils to tumor cells and convergence of granules to the EO-tumor cell synapses, suggesting degranulation upon cell contact. 

**Figure 5:**
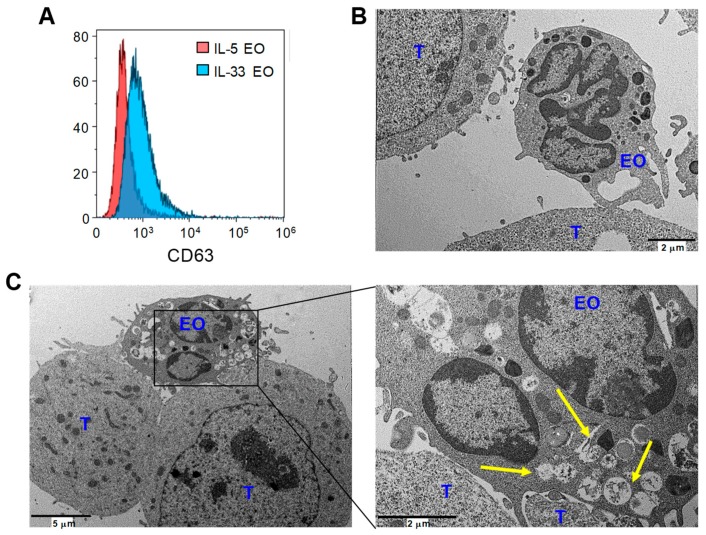
IL-33 increases eosinophil degranulation upon adhesion to tumor cells. (**A**) Flow cytometry analysis of CD63 surface membrane expression in IL-5 EO and IL-33 EO. Histograms from one out of three independent experiments are shown. (**B**,**C**) Ultrastructural analysis of IL-5 and IL-33 EO co-cultured with B16 tumor cells (T). (**B**) Representative image of IL-5 EO loosely bound to tumor cells with no evidence of granule content loss (electron-dense granules). (**C**) Representative IL-33 EO spreading elongations to take contact with two tumor cells. Enlargement shows emptying eosinophil granules with reduced electron density (indicated by arrows) in proximity to the cell contact site.

To better characterize the degranulation process in eosinophils, we evaluated the membrane expression of CD63, a marker up-regulated in activated eosinophils and strongly associated with secretory processes [34]. Flow cytometry analysis revealed higher surface levels of CD63 in IL-33 EO, compared with IL-5 EO (Figure 5A), indicating increased degranulation following IL-33 activation. Notably, polarized degranulation in IL-33 activated eosinophils upon contact with target tumor cells was clearly visible by TEM analysis. Here, IL-33 EO tightly bound to tumor cells and showed emptying granules, revealed by loss of electron density, in proximity of contact region (Figure 5C). In contrast, IL-5 EO loosely adhered to tumor cells and exhibited electron-dense granules, indicating no or little degranulation (Figure 5B).

**Figure 6:**
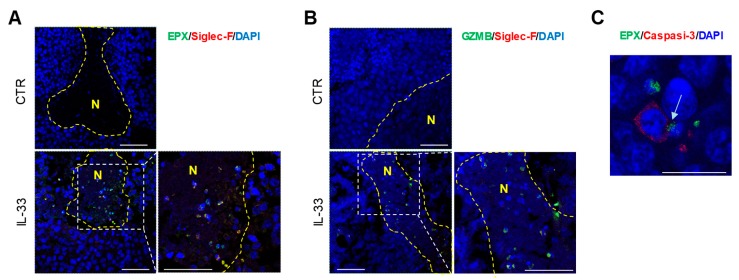
Detection of degranulating tumor-infiltrating eosinophils in mice treated with IL-33. CLSM analysis of tumor tissue sections from untreated (CTR) or IL-33 treated mice stained for EPX, Siglec-F, GZMB or Caspase-3 detection. Merged images (three-dimensional reconstruction) of (**A**) EPX/Siglec-F, (**B**) GZMB/Siglec-F and (**C**) EPX/Caspase-3 are reported. Co-localizations are shown in yellow. Inserts represent higher-power magnification images of necrotic areas (N) with EO infiltration in tissues from IL-33 treated mice. Nuclei are stained with DAPI (blue). Scale bars correspond to 50 µm (**A**,**B**) or 20 µm (**C**). Panels are representative of 2 independent experiments.

We further investigated the effects of IL-33 on granule convergence and degranulation of eosinophils in vivo in a mouse melanoma model. Treatment of mice implanted with B16 melanoma tumors with IL-33 results in tumor growth delay and accumulation of intratumoral eosinophils [6] (Figure S5). To determine whether these tumor-infiltrating eosinophils were cytotoxic, we analyzed eosinophil expression of cytolytic molecules in tumor tissues. As opposed to controls, tumor tissues from IL-33-treated mice displayed the substantial presence of degranulating tumor-infiltrating eosinophils within tumor necrotic areas, as revealed by co-expression of Siglec-F with EPX (Figure 6A, Figure S6) and granzyme-B (Figure 6B, Figure S7). Dual immunofluorescence staining of EPX and cleaved caspase-3 revealed juxtaposition of apoptotic tumor cells to intratumoral eosinophils with polarized EPX (arrow) in IL-33 treated mice (Figure 6C). As expected, tumor tissues from control mice showed rare tumor infiltrating eosinophils with no localization in tumor necrotic or caspase-3^+^ areas (Figure 6A,B, Figures S6 and S7, and data not shown).

### 2.4. Tumoricidal Functions of IL-33-Activated Eosinophils Require Cell Adhesion via CD11b/CD18

**Figure 7:**
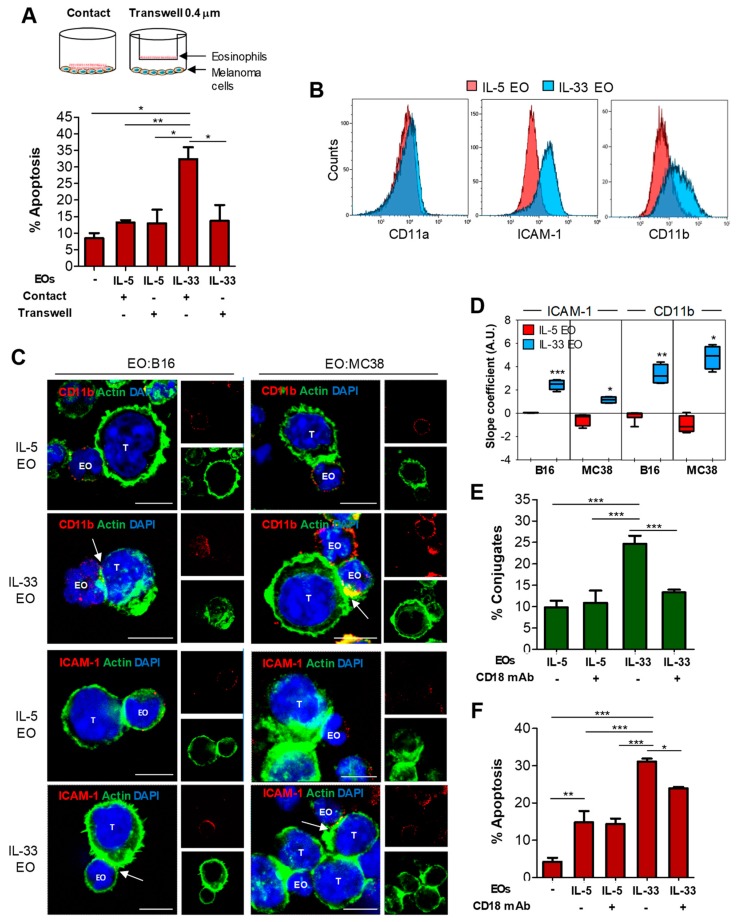
CD11b/CD18 mediates adhesion and tumor toxicity in IL-33 activated eosinophils. (**A**) Requirement of cell-cell contact for IL-33 EO-mediated cytotoxicity. B16 melanoma cells were co-cultured with IL-5 EO or IL-33 EO in contact or separated by a 0.4 µm Transwell insert. Tumor apoptosis was analyzed by flow cytometry. Mean of culture triplicates ± SD is shown. One representative experiment out of three. (**B**) Expression of the indicated adhesion molecules in IL-5 and IL-33 EO by flow cytometry. (**C**) CLSM examinations of IL-5 or IL-33 EO/target cell conjugates. Cell conjugates were stained for CD11b or ICAM-1 expression (red) and Alexa Fluor^®^-488 phalloidin for actin staining (green). DAPI was used to stain nuclei (blue). Merged images are reported and colocalization of adhesion proteins and actin at the immune synapse of EO/tumor cell conjugates is shown in yellow and depicted by arrows. Inserts represent separate channel images. Scale bars, 10 µm. Panels are representative of 4 independent experiments. (**D**) Quantitative determination of polarization of indicated molecules to the immune synapses by ImageJ software. Data are expressed as the mean polarization coefficient of several images depicting cell conjugates from 4 independent experiments ±SD. (**E**) Effect of blocking anti-CD18 mAb (10 µg/mL) on eosinophil adhesion to B16 melanoma cells, detected by flow cytometry. Data represent the mean percentage of EO-B16 conjugates ±SD (*n* = 3). (**F**) Effect of blocking anti-CD18 mAb on melanoma cell apoptosis after co-culture with IL-5 or IL-33 EO. Data represent the mean of Annexin-V^+^ tumor cells ± SD (*n* = 3). * *p* < 0.05, ** *p* < 0.01, *** *p* < 0.001.

The data described above suggest that IL-33 activates in eosinophils both adhesion to tumor cell target and granule convergence to immune synapses, leading to degranulation. To evaluate the actual requirement of cell adhesion for subsequent degranulation and tumor cell killing by eosinophils following IL-33 activation, eosinophils were cultured with tumor cells either in direct contact or separated by a 0.4 μm membrane (Transwell). The presence of a Transwell membrane between cells significantly abrogated tumor apoptosis caused by IL-33 EO, whereas it was irrelevant for IL-5 EO (Figure 7A), implying that cell contact was absolutely required for IL-33 induced tumoricidal functions of eosinophils.

In human eosinophils, cell adhesion and subsequent degranulation are mediated by integrins, such as CD11a/CD18 or CD11b/CD18 (Mac-1), and by intercellular adhesion molecule (ICAM)-1 [11,35,36]. Since IL-33 has been shown to up-regulate adhesion molecules in human eosinophils [26], we analyzed the expression of these proteins in IL-33 EO. IL-33 EO expressed higher levels of ICAM-1 and CD11b/CD18, but not CD11a/CD18, compared to IL-5 EO, as shown by flow cytometry (Figure 7B) and CLSM analysis (Figure S8). To evaluate the involvement of adhesion molecules in the formation of cell conjugates among eosinophils and tumor cells, we carried out CLSM of IL-33 and IL-5 EO in co-culture with tumor cells. CD11b/CD18 and, to a minor extent, ICAM-1 significantly polarized to the IL-33 EO-tumor cell synapses, whereas they were expressed evenly on the cell membrane of IL-5 EO conjugated with target tumor cells (Figure 7C,D). In contrast, we did not observe polarization of CD11a/CD18 to the EO-tumor cell synapses in either IL-33-activated or control IL-5 EO (Figure S9). These observations indicate a possible involvement of CD11b/CD18 and ICAM-1, but not CD11a, in the adhesion of IL-33-activated eosinophils to tumor cells.

Since CD11b/CD18 exhibited higher polarization magnitude than ICAM-1 (Figure 7D), we focused on CD11b/CD18 to evaluate its possible role in mediating contact-dependent cytotoxicity of IL-33 EO. For this purpose, we employed an anti-CD18 mAb to block the heterodimer CD11b/CD18, without activating degranulation that would otherwise be triggered by direct CD11b ligation [37]. Furthermore, we postulated that eventual blocking of CD11a/CD18 by this antibody would be functionally irrelevant since CD11a was not polarized in the EO-tumor cell synapses (Figure S9). Notably, co-culture of IL-33 EO with tumor cells in the presence of anti-CD18 blocking mAb significantly inhibited the formation of eosinophil-tumor cell conjugates (Figure 7E, Figure S10) and significantly reduced tumor cell killing (Figure 7F). In contrast, CD11b/CD18 blockade in IL-5 EO had no effects in either tumor cell adhesion (Figure 7E, Figure S10) or cytotoxicity (Figure 7F). Of interest, the few cell conjugates forming among IL-33 EO and tumor cells in the presence of anti-CD18 blocking mAb did not show increased ICAM-1 polarization (Figure S11), suggesting that ICAM-1 does not compensate for lack of CD11b/CD18-mediated adhesion. Thus, these results indicate that IL-33 activates adhesion dependent tumoricidal activities in eosinophils mainly through the integrin CD11b/CD18.

## 3. Discussion

The contradictory role eosinophils play in cancer may stem from the aberrant tumor microenvironment (TME), which can shape their phenotype and function, resulting in divergent responses. In this respect, eosinophils secrete a wide array of soluble mediators that can either promote tumorigenesis, angiogenesis, and metastasis or, inversely, halt tumor growth [2]. Accumulating evidence suggests that in vivo eosinophils may help control tumor growth and/or metastasis formation in models of melanoma [3,4,5,6], colorectal carcinoma [7], fibrosarcoma [9], hepatocellular and breast carcinoma [8,19]. We previously reported that IL-33 exerts anti-tumoral activities against melanoma growth and metastasis by facilitating the recruitment of eosinophils, which were crucial for therapeutic efficacy [6]. Tumor-infiltrating eosinophils may serve as accessory cells facilitating CD8 T cell recruitment to the TME through production of specific chemokines [5,6]. However, earlier evidence showing in situ eosinophil degranulation in tumor tissues from both mouse models [38] and cancer patients [39], suggested that eosinophils may be also capable of direct tumor cell killing. Indeed, in vitro killing of tumor cells by human [11,12,40] and mouse eosinophils [8,9] has been reported. Here, we confirm this concept and provide, for the first time, a mechanism induced by IL-33 and mediated by the integrin CD11b/CD18 that triggers granule convergence to immune cell synapses in eosinophil-tumor cell conjugates, degranulation, and tumor toxicity. 

We demonstrate that activation by IL-33 increases eosinophil cytotoxicity against several tumor cell lines via the induction of apoptosis. In keeping with this observation, other studies reported apoptosis as the principal tumor killing mechanism operated by eosinophils. In human eosinophils, the induction of apoptosis in colon cancer cells was shown to be mediated by granzyme-A and TNF-α [12]. Instead, in a study with murine hypodense eosinophils tumor apoptosis was mediated by granzyme-B [41]. Furthermore, ECP is also able to induce destruction of bronchial cells via caspase-8-dependent apoptosis [42] and it is believed to operate by altering the cell membrane permeability resulting in pore formation [43]. Of interest, IL-33 had no effect on eosinophil granule-protein expression, except for granzyme-B, which was more expressed in IL-33 EO than in IL-5 EO. This finding confirms our previous report showing that IL-33 increases the expression of granzyme-B in tumor-infiltrating eosinophils [6] and suggests that this molecule may be induced by IL-33 at transcriptional level. On the other hand, IL-33 may act on preformed granule stored cationic proteins EPX and ECP to regulate granule convergence to immune synapses, suggesting functional relevance for lytic granule secretion. Indeed, CLSM observations showed substantial polarization of granzyme-B and cationic proteins in IL-33 EO-tumor cell conjugates. These events led to eosinophil degranulation, as demonstrated by TEM analysis, showing emptying granules in IL-33 EO adjoining tumor cells, and by up-regulation of surface CD63, an intracellular granule marker that translocates to the cell membrane as a result of degranulation [34]. In natural killer (NK) cells, lytic granule convergence represents a mechanism by which these cells prepare to direct their cytotoxicity, avoiding non-specific multidirectional degranulation and off-target killing [44]. Thus, it is likely that eosinophils may operate through a similar mechanism and that IL-33 may facilitate both synapse formation and granule convergence, leading to targeted tumor cell killing.

Of note, our data indicate that IL-33 stimulates degranulation and cytotoxicity of eosinophils also in vivo. In fact, we observed substantial expression of granzyme-B and EPX in tumor-infiltrating EO induced by in vivo exposure to IL-33, at variance with control mice. Notably, in IL-33 treated mice we found tumor-infiltrating EO primarily in necrotic areas of melanoma tissue, with evidence of granule protein polarization towards tumor apoptotic cells. This observation is in agreement with previous reports showing eosinophil infiltration and release of granule proteins within necrotic and perivascular areas of murine tumors [38,45] and strongly suggests that IL-33-recruited eosinophils may be tumoricidal in vivo. In support of this view, we demonstrate that co-injection of IL-33 EO with melanoma cells delays tumor outgrowth in mice.

Induction of tumor apoptosis by IL-33-activated eosinophils was associated with the formation of increased numbers of conjugates with target tumor cells, demonstrated by flow cytometry and CLSM analysis, and was abrogated in the absence of cell contact. This finding is in accordance with previous reports showing contact-dependent tumor toxicity by human eosinophils [11,12] and supports the idea that adhesion molecules play a key role in activating eosinophil degranulation. In the attempt to identify possible molecules mediating adhesion of eosinophils to tumor cells, we focused on the integrins CD11a/CD18, CD11b/CD18, and ICAM-1. Although CD11a/CD18 was reported to mediate adhesion and subsequent killing of tumor cells by human eosinophils [12,46], this molecule was neither up regulated in IL-33 EO nor polarized to the EO-tumor cell synapses. Instead, IL-33 EO expressed increased levels of ICAM-1 and CD11b/CD18, and both molecules (mostly CD11b/CD18) polarized to the EO-tumor cell synapses. Strikingly, blocking of CD11b/CD18 in IL-33 EO significantly inhibited both tumor cell adhesion and killing, demonstrating a crucial role of this integrin in these processes. The observation that ICAM-1 did not compensate for lack of CD11b-mediated adhesion in EO-tumor cell conjugates upon CD11b/CD18 blockade further supports the major role played by CD11b/CD18 in mediating tumor cell adhesion and subsequent cytotoxicity in IL-33 EO. However, since after CD11b/CD18 blockade IL-33 EO were still able to induce higher levels of apoptosis compared to IL-5 EO, we cannot exclude the possibility that other adhesion molecules may participate in this process. 

Previous studies reported that IL-33 enhances both adhesion and CD11b/CD18 expression in human eosinophils, although the specific role of CD11b/CD18 in IL-33 induced eosinophil adhesion was not addressed [26]. CD11b/CD18 is reported to bind more than 30 ligands, including members of the ICAM family, extracellular matrix components such as fibrinogen, complement C3 fragment C3bi, and microbial ligands [47]. Therefore, the ligand(s) for CD11b/CD18 on tumor cells will require further investigation. Macrophages, neutrophils and NK cells exploit CD11b/CD18 for both adhesion and cytotoxic responses. Priming of CD11b/CD18 in circulating phagocytes and NK cells allows cytotoxic degranulation in response to iC3b-opsonized tumor cells [48]. In macrophages, exposure to GM-CSF induced the expression of CD11b/CD18 and increased the attachment to tumor cells, resulting in efficient contact-dependent tumor cell lysis [49]. Furthermore, CD11b/CD18 was essential for antibody-dependent cellular cytotoxicity (ADCC) and immunologic synapse formation toward tumor targets in neutrophils [50] and in eosinophils against parasites [51]. In human eosinophils, engagement of CD11b/CD18 by ligation with an anti-CD11b mAb activates an intracellular signaling cascade, involving protein tyrosine phosphorylation and phosphoinositide hydrolysis, causing degranulation [37]. Thus, CD11b/CD18 may actively participate in the intracellular pathways leading to functional activation of eosinophils and IL-33 may serve as a trigger for CD11b/CD18 signaling cascade.

IL-33 drives substantial eosinophilia in vivo [23,24], although it is unclear whether this effect occurs via a direct or indirect mechanism. Since IL-33 is an alarmin and because alarmins may act as chemoattractants, such as HMGB1 [52], we wanted to explore this possibility. Our migration assays with microfluidic chips and transwell chambers demonstrate that IL-33 is not a direct chemoattractant for eosinophils. In fact, IL-33 promoted eosinophil migration to the tumor indirectly, via induction of the chemokines CCL5 and CCL24/eotaxin-2 by tumor cells themselves. This data is noteworthy and indicates that, within the TME, tumor cells are a source of eosinophil-attracting chemokines. 

Overall, our study provides mechanistic insights into how IL-33 may induce eosinophil recruitment and anti-tumoral activities within the TME. From a physiological point of view, eosinophils may perceive danger signals and alarmins, such as IL-33, released from necrotic or stressed cells within the TME and get activated [53]. On the other hand, tumor expression of IL-33 may induce the production of chemokines that recruit eosinophils [6]. In this view, expression of IL-33 has been reported in several human tumors, although its correlation with tumor progression is controversial [15]. Nevertheless, tumor-associated and blood eosinophilia are emerging as potential biomarkers predictive of tumor progression, clinical outcome and therapy response [2]. Elevated counts of peripheral blood eosinophils are associated with improved survival in advanced melanoma patients undergoing immunotherapy with the immune checkpoint inhibitors ipilimumab or pembrolizumab [54,55,56]. Furthermore, eosinophils participate in immune checkpoint inhibitor-based immunotherapy in pre-clinical tumor models [19]. In this scenario, the TME plays a pivotal role in shaping the anti-tumorigenic properties of eosinophils. Reichman et al. suggested that local concentration of IFN-γ may polarize eosinophils towards an anti-tumorigenic phenotype [7]. We propose that IL-33 may be one pro-inflammatory alarmin “natural allied” for eosinophils within the TME promoting their recruitment and activating their tumoricidal functions via cell adhesion and targeted degranulation. Future therapeutic directions should take into account this axis for more effective strategies against cancer.

## 4. Materials and Methods

### 4.1. Tumor Cell Lines and Cytokines

Murine B16.F10 metastatic melanoma cells (ATCC, CRL-6475), MC38 colon carcinoma cells (kindly provided by Dr. Carlos Alfaro, University of Navarra, Pamplona, Spain), TC-1 lung carcinoma cells (kindly provided by Dr. Guido Kroemer, Gustave Roussy Cancer Institute, Villejuif, France) and MCA205 fibrosarcoma cells (Merck Millipore, Burlington, MA, USA, SCC173) were used. All cell lines were routinely tested for morphology, growth curve and absence of mycoplasma and passaged for no more than 4 times from thawing. Recombinant mouse IL-33 carrier-free (amino acids Ser109-Ile266) was purchased from Biolegend (San Diego, California, USA). The 158 amino acid recombinant protein has a predicted molecular mass of approximately 17,554 Da. The DTT-reduced and non-reduced protein migrates at approximately 20 kDa by SDS-PAGE. The N-terminal amino acid is Serine. Recombinant mouse IL-5, rm GM-CSF, and rm SCF were purchased from Peprotech (London, UK), while rm FLT3-L was obtained from Milteny Biotec (Bergisch Gladbach, Germany).

### 4.2. Differentiation of Bone Marrow-Eosinophils 

Eosinophils were generated from cultures of bone marrow cells following a protocol previously described [6]. Briefly, bone marrow cells from naïve C57Bl/6 mice were cultured at 1 × 10^6^/mL in RPMI 1640 (EuroClone, Pero, Milan, Italy) containing 20% FBS, 1% glutamine, 25 mM Hepes, 1X NEAA, 1 mM sodium pyruvate, supplemented with 100 ng/mL SCF and 100 ng/mL FLT3-L. From day 4 to day 10, 10 ng/mL IL-5 was added to the culture every other day. From day 10, either IL-5 or IL-33 (100 ng/mL) were added every other day in order to generate IL-5 eosinophils (IL-5 EO) and IL-33 eosinophils (IL-33 EO). Cells were used on day 15 or 16, after 24 h incubation with 10 ng/mL of GM-CSF. Eosinophil purity (>80%) was determined by flow cytometry (CD11b^+^Siglec-F^+^Ly6G^−^CD11c^−^) and by cytospin.

### 4.3. Migration Assay with Microfluidic Devices 

Microfluidic devices for co-culture competitive assay were fabricated in polydimethylsiloxane (PDMS), as previously described [31]. Briefly, the master molds were created by a two-layer microfabrication process, using the negative photoresist SU-8 (MicroChem Corp, Newton, MA, USA) and carried out according to parameters specified in the manufacturer’s datasheets. Patterns for standard photolithography were designed with CAD software and transferred onto two chrome masks by electron-beam lithography. B16 melanoma cells were stained with PKH67 Green Fluorescent Cell Linker (Sigma-Aldrich, St. Louis, MO, USA) and resuspended in Matrigel (2 mg/mL, BD Biosciences, San Jose, CA, USA). Where indicated, IL-33 (100 ng/mL, Biolegend) was added to the cell-Matrigel mixture. The B16 cell-Matrigel mixtures (2 × 10^4^ cells in 3 µL) were then loaded in the narrow gel chambers devices. After B16 cell-Matrigel loading, the device was put at 37 °C for 30 min to allow gel solidification. In a second step, standard bone marrow-derived eosinophils (IL-5 EO) were labeled with the PKH26 Red Fluorescent Cell Linker (Sigma). Eosinophils were resuspended in culturing medium and loaded (1 × 10^6^ cells in 200 µL) in the central chamber of the device, while lateral reservoir chambers were filled with medium. The devices were placed in a 37 °C, 5% CO_2_ incubator. Phase-contrast, visible and fluorescence photomicrographs were generated at various times by using an EVOS-FL fluorescence microscope (Life Technologies, Carlsbad, CA, USA), provided with built-in imaging software for image overlays. Fluorescence analysis was performed using ImageJ software (National Institutes of Health. Bethesda, MD, USA). 

### 4.4. Transwell Migration Assay

Transwell migration assay of eosinophils was performed using 5 μm pore-sized cell culture inserts (Corning Costar Corporation, Cambridge, MA). Briefly, B16 melanoma cells were seeded (2 × 10^4^ in 600 µL) in the bottom compartment with or without IL-33 (100 ng/mL). Alternatively, the bottom compartment was filled with 600 µL of 2% FCS DMEM medium alone or containing IL-33 (100 ng/mL) or CCL11 (100 ng/mL, Biolegend). Eosinophils (IL-5 EO) were seeded in the upper insert (2 × 10^5^ in 100 µL). After 18 h incubation at 37 °C, 5% CO_2_ the eosinophils migrated in the bottom compartment were enumerated by means of Neubauer chambers.

### 4.5. Chemokine Expression by qPCR

B16.F10 melanoma cells were cultured in the presence or absence of IL-33 (100 ng/mL) for 4 h. Total RNA was extracted from tumor cells by using TRIsure reagent (Bioline, London, UK). mRNA was reverse transcribed by means of Tetro cDNA Synthesis Kit (Bioline). Quantitative reverse transcription-PCR (qPCR) with forward and reverse primers for CCL5, CCL24 and HPRT (Eurofins Genomics, Ebersberg, Germany) [6] was performed using Sensimix Plus SYBR Kit containing the fluorescent dye SYBR Green (Bioline) and by means of an ABI 7500 Real-time PCR system (Applied Biosystems, Thermo Fisher Scientific, Waltham, Ma, USA). Triplicates were performed for each experimental point. Data were normalized to HPRT (2-ΔCt method) and presented as fold change expression vs. control.

### 4.6. Flow Cytometry

Phenotypic analysis of eosinophils. Bone marrow-derived IL-5 EO and IL-33 EO were stained with the following fluorescently labeled mAbs from BD Biosciences, Biolegend or Thermo Fisher: anti-CD11a/CD18 (H155-78), anti-CD11b/CD18 (M1/70), anti-CD11c (N418), anti-CD45 (30-F11), anti-CD63 (NVG-2), anti-CD69 (H1.2F3), anti-Ly6G (1A8), anti-Siglec-F (E50-2440). Samples were run on a Gallios flow cytometer and analyzed with the Kaluza Analysis Software (Beckman Coulter, Pasadena, CA, USA). Tumor infiltrating eosinophils from B16 melanoma-bearing mice (see below) were identified as previously described [6]. Briefly, tumors were digested in DNase I (325 KU/mL, Sigma) and type III collagenase (1 mg/mL, Worthington Biochemical Corporation, Lakewood, NJ, USA) containing medium, passed through a cell sieve and the resulting cell suspension was subjected to lysis of erythrocytes in 140 mM NH4Cl, 17 mM Tris HCl, pH 7.2. Cells were then stained with the fluorescently labeled mAbs to identify tumor-infiltrating eosinophils (CD45^+^CD11b^hi^Siglec-F^hi^Ly6G^−^).

### 4.7. Tumor Cell-Adhesion Assay

Adhesion of eosinophils to tumor cells was assessed by flow cytometry, as previously described [11,12]. Briefly, tumor cells were labeled with PKH26 Red fluorescent Cell Linker (Sigma) and then co-cultured for 1 h with IL-5 or IL-33 EOs at 10:1 EO:tumor cell ratio. In some experiments, eosinophils were incubated with 10 µg/mL anti-CD18 blocking mAb (M18/2, Biolegend) for 20 min at 4 °C prior co-culture with tumor cells. Cells were then stained with BV421 anti-Siglec-F mAb and analyzed by flow cytometry. The percentage of cell conjugates was determined as follows: fraction of Siglec-F^+^ PKH26^+^/total PKH26^+^ tumor cells × 100.

### 4.8. Cytotoxicity Assay

Eosinophil-mediated cytotoxicity against tumor cells was evaluated as previously described [12]. Briefly, tumor cells were labeled with the PKH26 Red fluorescent Cell Linker (Sigma) and then seeded in 96 wells U-bottomed plates (1 × 10^4^ cells per well) in the presence of eosinophils (IL-5 EO or IL-33 EO) at different E:T ratios. Where indicated, eosinophils were pre-incubated with 10 µg/mL anti-CD18 blocking mAb for 20 min at 4 °C. Co-cultures were incubated for 5 h at 37 °C. Cells were then stained with Annexin-V (e-Bioscience, Thermo Fisher Scientific) and then analyzed by flow cytometry. Apoptosis of target tumor cells was calculated as the percentage of Annexin-V^+^ cells among gated PKH26^+^ population. For contact inhibition experiments, PKH26-labeled tumor cells were seeded (10^5^ in 500 µL) in 24 well plates and allowed to adhere for 1 h at 37 °C, 5% CO_2_. Eosinophils (2 × 10^6^ in 100 µL) were then added either directly to the wells or to a 0.4 µm pore-sized Transwell insert placed on the wells. Cells were incubated at 37 °C, 5% CO_2_ for 5 h and then stained with Annexin-V for determination of tumor apoptosis.

### 4.9. Analysis of Eosinophil-Tumor Cell Interactions by Time-Lapse Video Microscopy

Eosinophils (3 × 10^5^ cells) were co-cultured with tumor cells (B16 or TC-1, 10^5^ cells) in a 25-cm^2^ flask. Time lapse recordings were performed over a period of 24 h (1 microphotograph every 3 min) by means of a JuLI Smart microscope (Bulldog Bio, Inc., Portsmouth, NH, USA) placed directly inside the CO2 incubator for all the duration of the recording. Tracking analysis of time-lapse microphotographs was performed by using the ImageJ plugin TrackMate (https://imagej.net/TrackMate) in several 200 × 200 pixel video crops. Each crop is defined by the presence of a centered target tumor cell and the tracked eosinophils close to this target cell. Interaction times between eosinophils and tumor cells were calculated, as previously described [29]. In some experiments, at the end of the 24 h co-culture time, eosinophils were washed out with PBS. The remaining adherent tumor cells were fixed with methanol, stained with 0.1% Crystal Violet (in a 20% ethanol solution) and observed under a microscope. Quantification of tumor cells surviving to eosinophil-mediated killing was performed using ImageJ software.

### 4.10. Co-Culture of Eosinophils with Tumor Spheroids

Multicellular tumor spheroids from B16 melanoma cells were generated in ultralow-attachment surface 96-well plates (Corning Inc., Corning, NY, USA). One hundred cells were cultured in 200 μL of DMEM supplemented with 10% FBS, 1% penicillin-streptomycin-fungizone and 1% glutamine. Eosinophils (either IL-5 EO or IL-33 EO) were added (2.5 × 10^3^) to tumor cells from day 0, in order to evaluate their effects on 3D spheroid formation. Images were obtained from day 2 to day 9 of culture by using a visible light microscope.

### 4.11. Confocal Laser Scanning Microscopy (CLSM)

Eosinophils were co-cultured with B16 or MC38 cells (5:1 ratio) for 60 min at 37 °C and then transferred onto poly-L-lysine-coated coverslips. Detection of adhesion molecules was performed by staining cell conjugates at 4 °C with biotin-conjugated mAbs anti-CD11b/CD18 (M1/70, BD Bioscences), anti-ICAM-1 (30F11, BD Bioscences), followed by streptavidin-Alexa Fluor-594 secondary Abs (Thermo Fisher Scientific). CD11a was detected by rat anti mouse CD11a/CD18 (H155-78; Biolegend), followed by goat anti-rat Alexa Fluor-594 secondary Ab (Thermo Fisher Scientific). Cells were then fixed by paraformaldehyde 3% and permeabilized by 0.5% Triton X-100, followed by actin staining with Alexa Fluor^®^-488 or 594-conjugated phalloidin (Thermo Fisher Scientific). For granule protein intracellular staining, FITC-conjugated EPX (Biorbyt, Cambridge, UK), FITC-conjugated granzyme-B (eBioscience) and unconjugated ECP (Biorbyt), followed by goat anti-rabbit Alexa Fluor^®^-488 secondary Ab (Thermo Fisher Scientific), were used. Cover glasses were finally mounted on microscope slides with Vectashield antifade mounting medium containing DAPI (Vector Laboratories, Burlingame, CA, USA). In some experiments, prior co-culture, EO and target cells were stained with the membrane dyes PKH26 and PKH67 (Sigma), respectively, according to manufacturer instructions. In experiments with anti-CD18 antagonist, EO were first incubated with anti-CD18 mAb (10 µg/mL) for 20 min at 4 °C, then mixed with target cells and allowed to conjugate for 60 min at 37 °C. CLSM observations were performed with a Leica TCS SP2 AOBS apparatus, using a 63×/1.40 NA oil objective and excitation spectral laser lines at 405, 488 and 594 nm. Image acquisition and processing were carried out using the Leica Confocal Software 2.6 rel 1537 (Leica Microsystems, Wetzlar, Germany) and Adobe Photoshop CS5 software programs (Adobe Systems Inc, San Jose, CA, USA). Signals from different fluorescent probes were taken in sequential scan settings. Several cell conjugates for each labeling condition were analyzed and representative results are shown. Quantification of signals at immunological synapse was obtained by means of ImageJ software and polarization was expressed as slope values (Figure S12).

### 4.12. Transmission Electron Microscopy (TEM)

IL-5 and IL-33 EO were co-cultured with B16 melanoma cells (10:1 ratio) for 60 min at 37 °C and then fixed in 2.5% glutaraldehyde in 0.1 M cacodylate buffer, pH 7.4. Following washes in cacodylate buffer, cells were post-fixed in 1% OsO_4_ in the same buffer and further washed with 0.1 M cacodylate. Cells were then dehydrated in ethanol gradient from 50% to 100% (*v*/*v*) and embedded in Agar 100 resin (Agar Scientific, Essex, UK) at 65 °C for 48 h. Ultrathin sections were obtained using an ultra-microtome and collected on 200-mesh grids, counterstained with uranyl acetate for 10 min and lead citrate for further 10 min. Samples were observed in a Philips 208s transmission electron microscope at 100 kW (Philips, Amsterdam, The Netherlands).

### 4.13. Mice and In Vivo Studies

C57BL/6 mice were purchased from Charles River Laboratories Italia (Calco, Lecco, Italy). All procedures were approved by the Italian Ministry of Health (Authorization no. 243/2016-PR; Protocol no. D9997.13) in accordance with EU regulations. For the detection of intratumoral eosinophils, female C57BL/6 mice were injected subcutaneously with 0.8 × 10^6^ B16.F10. Recombinant mouse IL-33 (0.4 μg per mouse), dissolved in 200 μL of phosphate-buffered saline (PBS), was injected intraperitoneal in mice 5 times, every other day, starting from day 6/7, when mean tumor diameter was 3 mm. Control groups consisted of mice injected with PBS. Mice were sacrificed 3 days after the last IL-33 administration for tumor explant. For evaluation of in vivo tumor cytotoxicity of eosinophils, C57BL/6 mice were injected subcutaneously with 0.8 × 10^6^ B16.F10 alone or mixed with eosinophils (either IL-5 EO or IL-33 EO, 10 × 10^6^ cells/mouse) or with naïve syngeneic splenocytes (10 × 10^6^ cells/mouse) as a control. Tumor growth was monitored using a digital caliper.

### 4.14. Immunofluorescence of Tumor Tissues

Formalin-fixed paraffin-embedded tissue sections (5 µm thick) were deparaffinized, hydrated through graded alcohols and subjected to a heat-induced epitope retrieval step by Tris-EDTA pH 7.2 for 3 × 3 min in a microwave oven. Sections were washed with 0.01% Tween 20 in PBS and then blocked with 3% BSA in PBS for 60 min at 37 °C. For analysis of cytotoxic eosinophils, Siglec-F expression was detected by staining with a rat anti-mouse primary mAb (BD Bioscences) for 30 min at 37 °C followed by goat anti-rat Alexa Fluor^®^-543 secondary Ab plus DAPI (Thermo Fisher Scientific). Granzyme-B and EPX detection were performed with the same Abs described above, and cleaved caspase-3 was detected with a rabbit anti-mouse Ab (Cell Signaling Technology, Inc., Danvers, MA, USA) followed by goat anti-rabbit Alexa Fluor^®^-594 secondary Abs (Thermo Fisher Scientific) plus DAPI. Immunofluorescence signals were acquired by CLSM observations.

### 4.15. Statistical Analysis

One-way ANOVA analysis of variance was performed to compare means among multiple groups, followed by post hoc testing (Tukey). Mann-Whitney test was used for the nonparametric analysis of differences between two groups. Values were considered significant when the probability was below the 5% confidence level (*p* < 0.05).

## 5. Conclusions

Our study unravels novel mechanisms for eosinophil-mediated antitumor activities following immunotherapy with IL-33 in mice. This cytokine recruits eosinophils to the tumor sites indirectly, through induction of tumor-derived chemokines, while it activates eosinophils directly promoting CD11b/CD18-mediated adhesion to tumor cells and lytic granule convergence to the immune synapse, leading to efficient tumor cell killing in vitro and in vivo. Therapeutic strategies enabling tumor infiltration and proper activation of eosinophils may improve the response in cancer patients.

## Supplementary Materials

The following are available online at https://www.mdpi.com/2072-6694/11/11/1664/s1, Figure S1: Images from Figure 2E, Figure S2: Effect of IL-33 EO vs. IL-5 EO on tumor spheroids, Figure S3: Interactions between eosinophils and TC-1 tumor cells, Figure S4: Expression of EPX, ECP and GZMB in eosinophils by CLSM, Figure S5: Recruitment of tumor-infiltrating eosinophils following IL-33 exposure in vivo. Figure S6: Single fluorescence signals from Figure 6A. Figure S7: Single fluorescence signals from Figure 6A; Figure S8: Expression of CD11b and ICAM-1 in eosinophils by CLSM; Figure S9: CD11a does not polarize to the EO-tumor cell synapses; Figure S10: Inhibition of EO/tumor cell conjugates by CD11b/CD18 blockade by CLSM; Figure S11: ICAM-1 does not compensate for the lack of CD11b/CD18-mediated adhesion. Figure S12: Quantitative analysis of protein polarization to the immune synapses. Video S1: IL-5 EO co-cultured with B16 tumor cells; Video S2: IL-33 EO co-cultured with B16 tumor cells; Video S3: IL-5 EO co-cultured with TC-1tumor cells; Video S4: IL-33 EO co-cultured with TC-1 tumor cells.
