# Supplementary material for: IL-33 Promotes CD11b/CD18-Mediated Adhesion of Eosinophils to Cancer Cells and Synapse-Polarized Degranulation Leading to Tumor Cell Killing
Source: Cancers (Basel). 2019 Oct 26;11(11):1664. doi: 10.3390/cancers11111664 (PMC6895824; doi:10.3390/cancers11111664)
Supplement: Supplementary file 1 [file cancers-11-01664-s001.zip › cancers-614811-suppl figures.pdf]

Supplementary Video Legends

Video 1. Video recording of IL-5 EO co-cultured with B16 tumor cells. The video (200 × 200 micron crop) shows tracks of IL-5 EO moving nearby a tumor cell (5 h time lapse). Colored lines depict tracks of IL-5 EO paths. Adherent cell in the middle of the field represents a B16 tumor cell.

Video 2. Video recording of IL-33 EO co-cultured with B16 tumor cells. The video (200 × 200 micron crop) shows tracks of IL-33 EO moving nearby a tumor cell (5 h time lapse). Colored lines depict tracks of IL-33 EO paths. Adherent cell in the middle of the field represents a B16 tumor cell.

Video 3. Video recording of IL-5 EO co-cultured with TC-1 tumor cells. The video (200 × 200 micron crop) shows tracks of IL-5 EO moving nearby a tumor cell (5 h time lapse). Colored lines depict tracks of IL-5 EO paths. Adherent cell in the middle of the field represents a TC-1 tumor cell.

Video 4. Video recording of IL-33 EO co-cultured with TC-1 tumor cells. The video (200 × 200 micron crop) shows tracks of IL-33 EO moving nearby a tumor cell (5 h time lapse). Colored lines depict tracks of IL-33 EO paths. Adherent cell in the middle of the field represents a TC-1 tumor cell.
