# Supplementary material for: IL-33 Promotes CD11b/CD18-Mediated Adhesion of Eosinophils to Cancer Cells and Synapse-Polarized Degranulation Leading to Tumor Cell Killing
Source: Cancers (Basel). 2019 Oct 26;11(11):1664. doi: 10.3390/cancers11111664 (PMC6895824; doi:10.3390/cancers11111664)
Supplement: Supplementary file 1 [file cancers-11-01664-s001.zip › cancers-614811-suppl figures.pptx]

## Slide 1
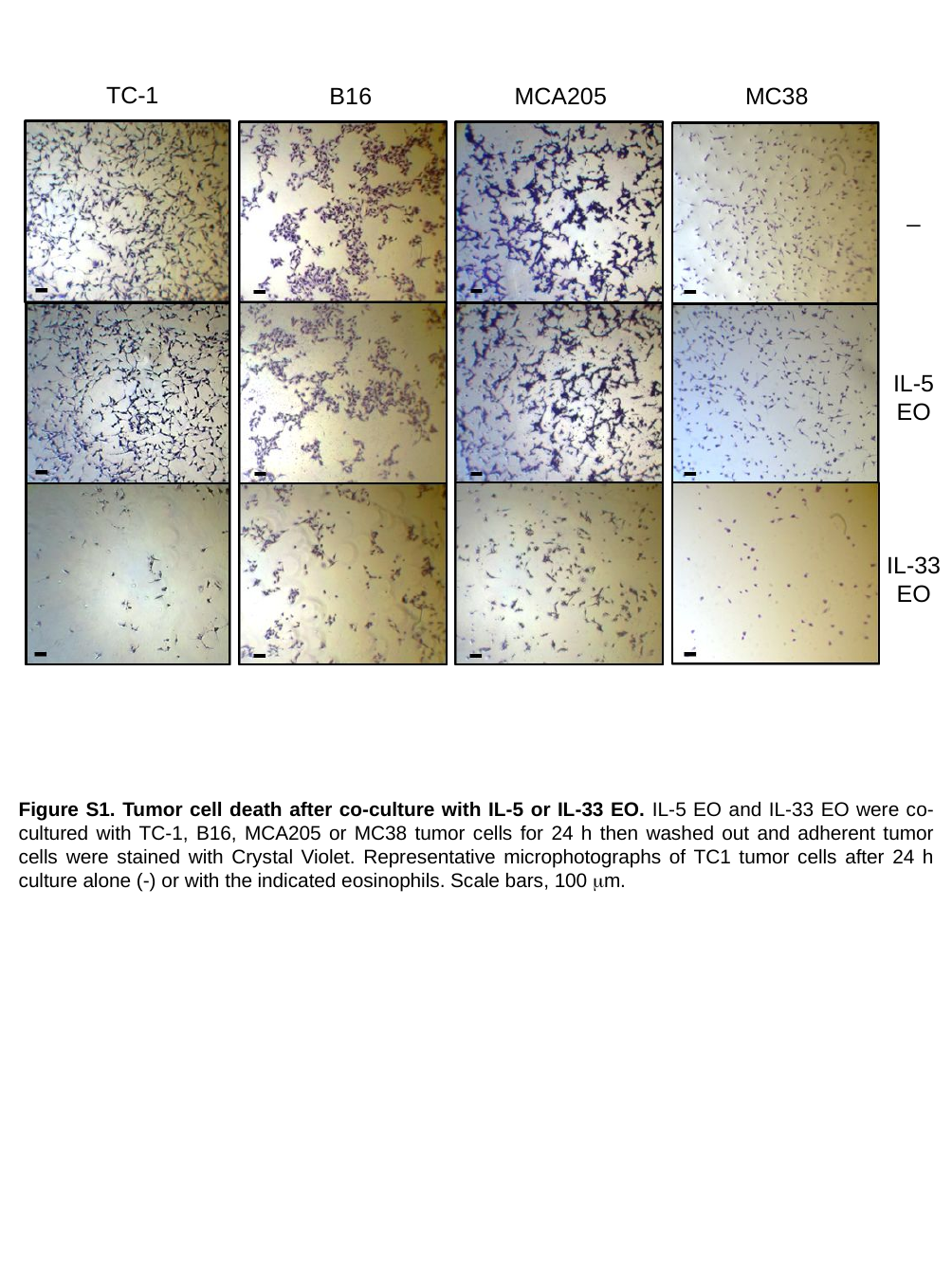

TC-1
B16
MCA205
MC38
_
IL-5
EO
IL-33
EO
Figure S1. Tumor cell death after co-culture with IL-5 or IL-33 EO. IL-5 EO and IL-33 EO were co-cultured with TC-1, B16, MCA205 or MC38 tumor cells for 24 h then washed out and adherent tumor cells were stained with Crystal Violet. Representative microphotographs of TC1 tumor cells after 24 h culture alone (-) or with the indicated eosinophils. Scale bars, 100 μm.

## Slide 2
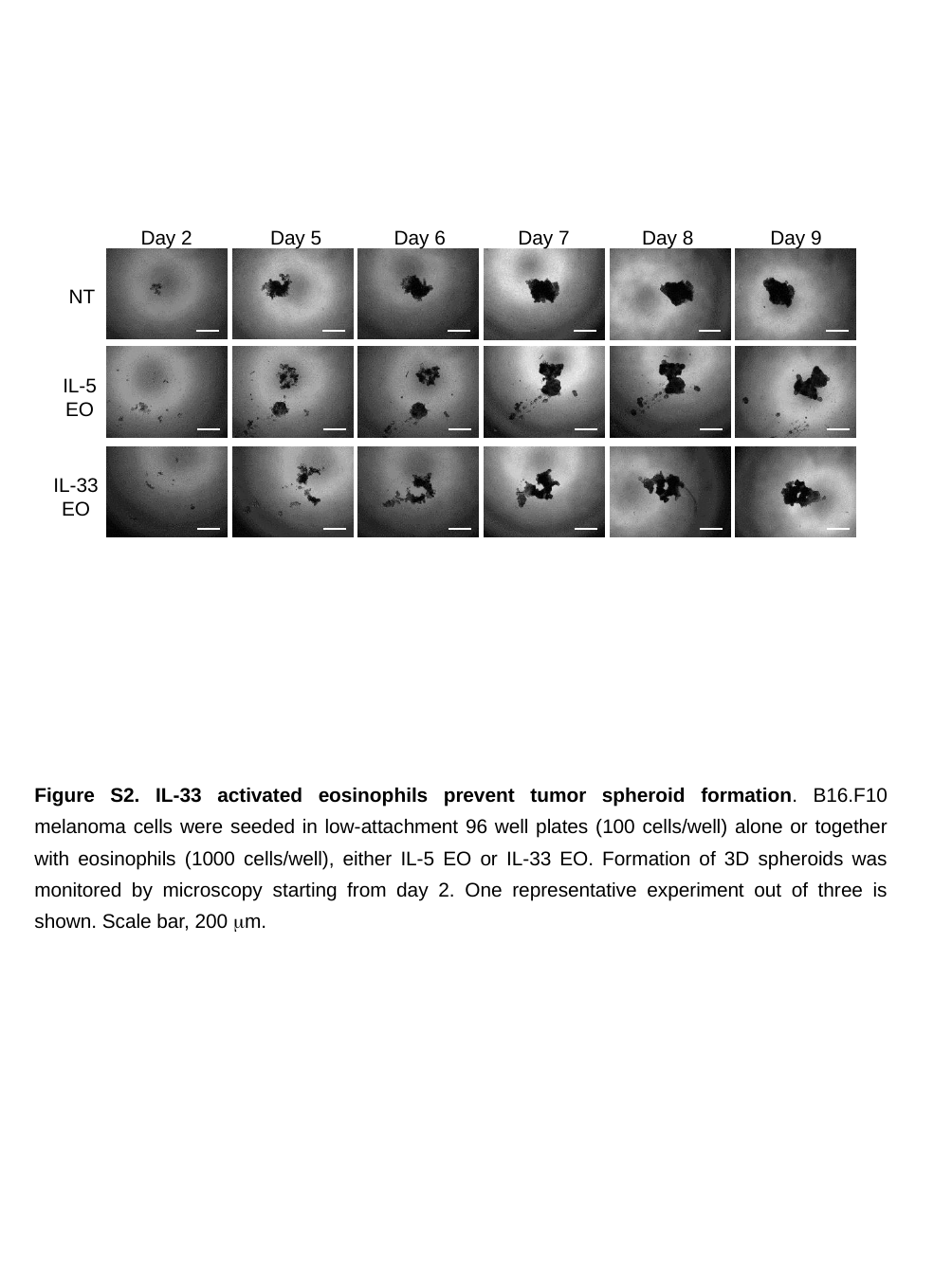

Day 2
Day 5
Day 6
Day 7
Day 8
Day 9
NT
IL-5
EO
IL-33
EO
Figure S2. IL-33 activated eosinophils prevent tumor spheroid formation. B16.F10 melanoma cells were seeded in low-attachment 96 well plates (100 cells/well) alone or together with eosinophils (1000 cells/well), either IL-5 EO or IL-33 EO. Formation of 3D spheroids was monitored by microscopy starting from day 2. One representative experiment out of three is shown. Scale bar, 200 μm.

## Slide 3
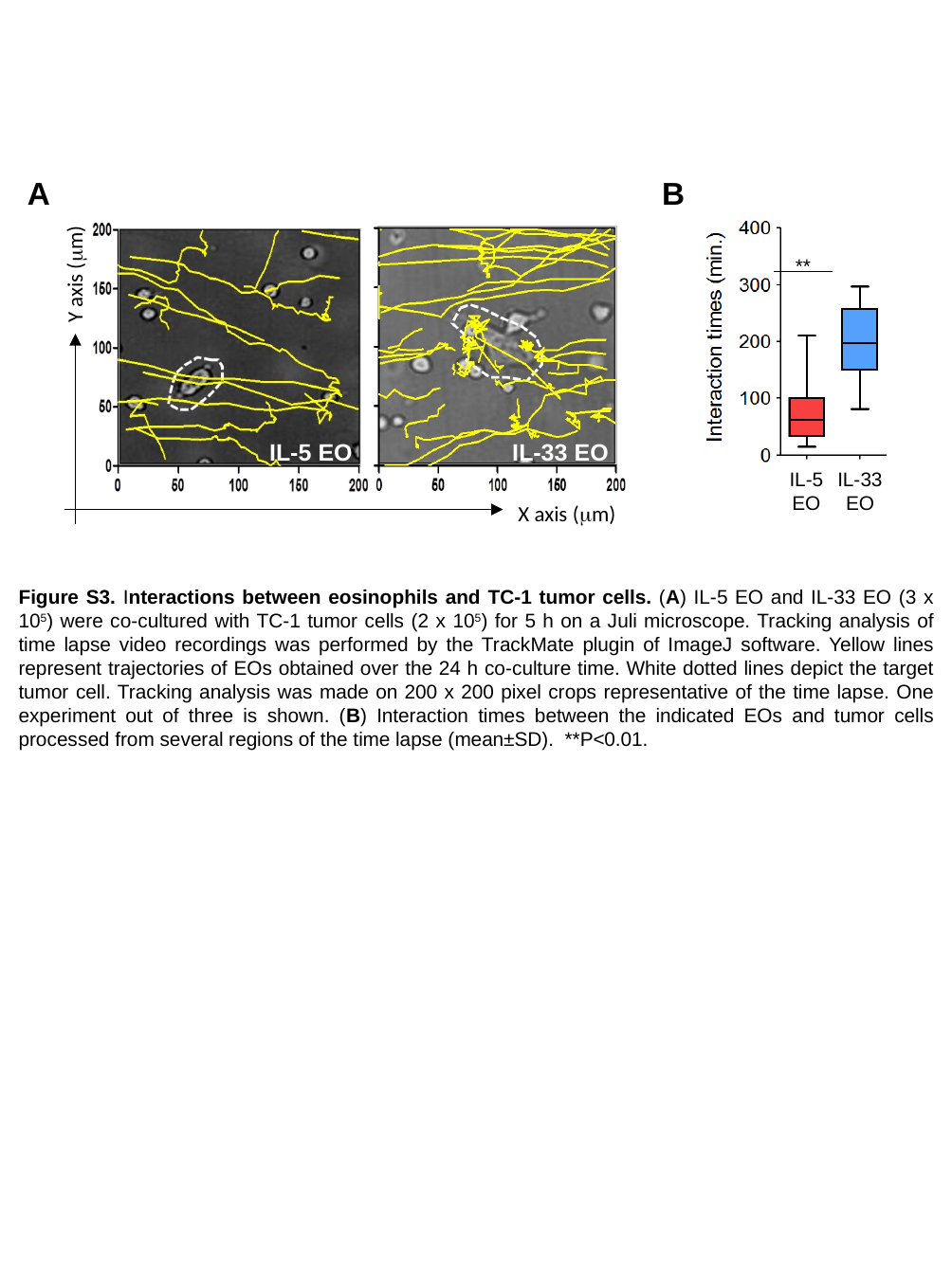

A
B
IL-33 EO
IL-5 EO
IL-5
EO
IL-33
EO
**
Y axis (mm)
X axis (mm)
Figure S3. Interactions between eosinophils and TC-1 tumor cells. (A) IL-5 EO and IL-33 EO (3 x 10^5^) were co-cultured with TC-1 tumor cells (2 x 10^5^) for 5 h on a Juli microscope. Tracking analysis of time lapse video recordings was performed by the TrackMate plugin of ImageJ software. Yellow lines represent trajectories of EOs obtained over the 24 h co-culture time. White dotted lines depict the target tumor cell. Tracking analysis was made on 200 x 200 pixel crops representative of the time lapse. One experiment out of three is shown. (B) Interaction times between the indicated EOs and tumor cells processed from several regions of the time lapse (mean±SD). **P<0.01.

## Slide 4
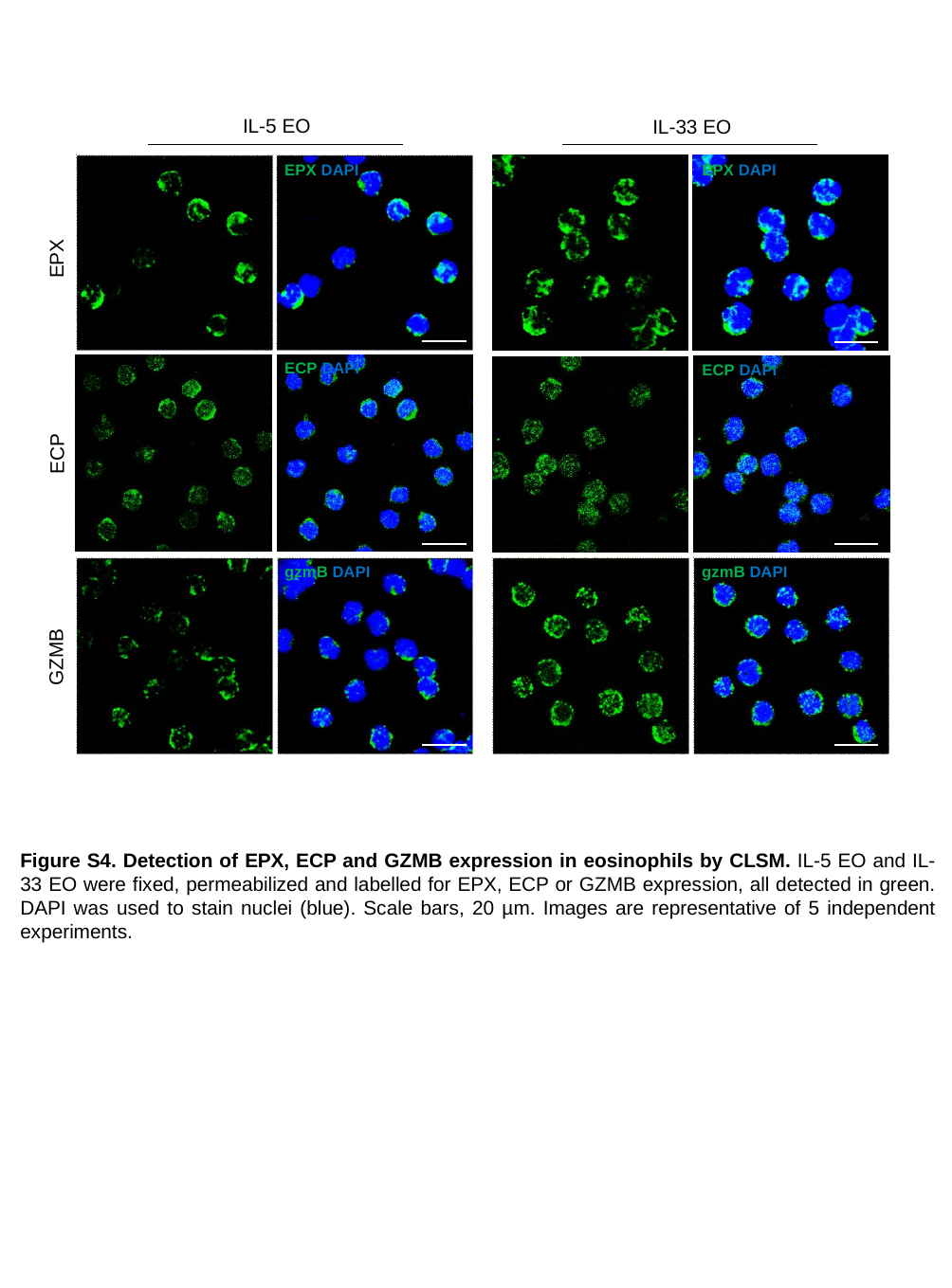

IL-5 EO
IL-33 EO
EPX/DAPI
EPX/DAPI
EPX
ECP/DAPI
ECP/DAPI
ECP
gzmB/DAPI
gzmB/DAPI
GZMB
Figure S4. Detection of EPX, ECP and GZMB expression in eosinophils by CLSM. IL-5 EO and IL-33 EO were fixed, permeabilized and labelled for EPX, ECP or GZMB expression, all detected in green. DAPI was used to stain nuclei (blue). Scale bars, 20 µm. Images are representative of 5 independent experiments.

## Slide 5
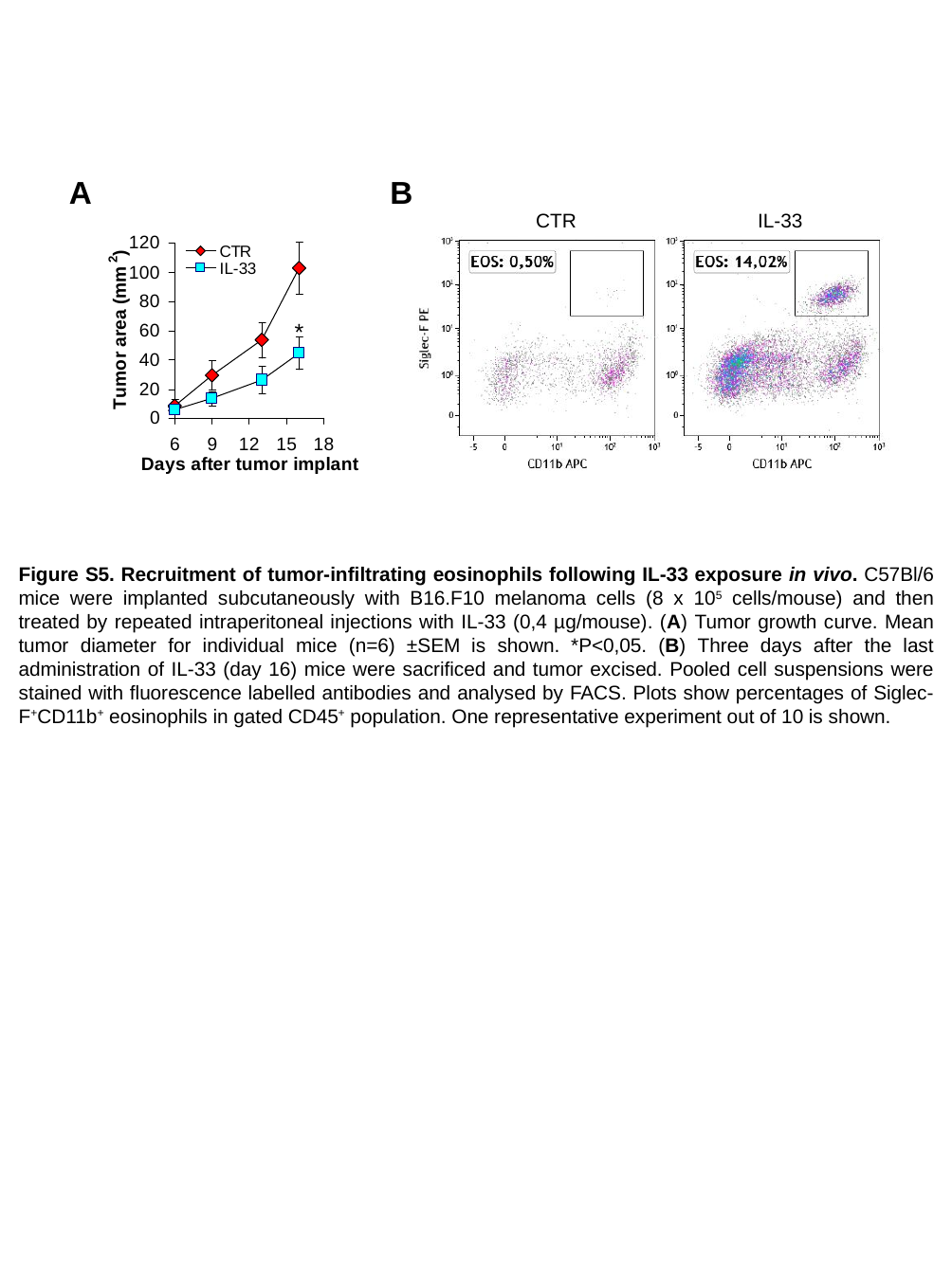

A
B
CTR
IL-33
*
Figure S5. Recruitment of tumor-infiltrating eosinophils following IL-33 exposure in vivo. C57Bl/6 mice were implanted subcutaneously with B16.F10 melanoma cells (8 x 10^5^ cells/mouse) and then treated by repeated intraperitoneal injections with IL-33 (0,4 µg/mouse). (A) Tumor growth curve. Mean tumor diameter for individual mice (n=6) ±SEM is shown. *P<0,05. (B) Three days after the last administration of IL-33 (day 16) mice were sacrificed and tumor excised. Pooled cell suspensions were stained with fluorescence labelled antibodies and analysed by FACS. Plots show percentages of Siglec-F+CD11b+ eosinophils in gated CD45+ population. One representative experiment out of 10 is shown.

## Slide 6
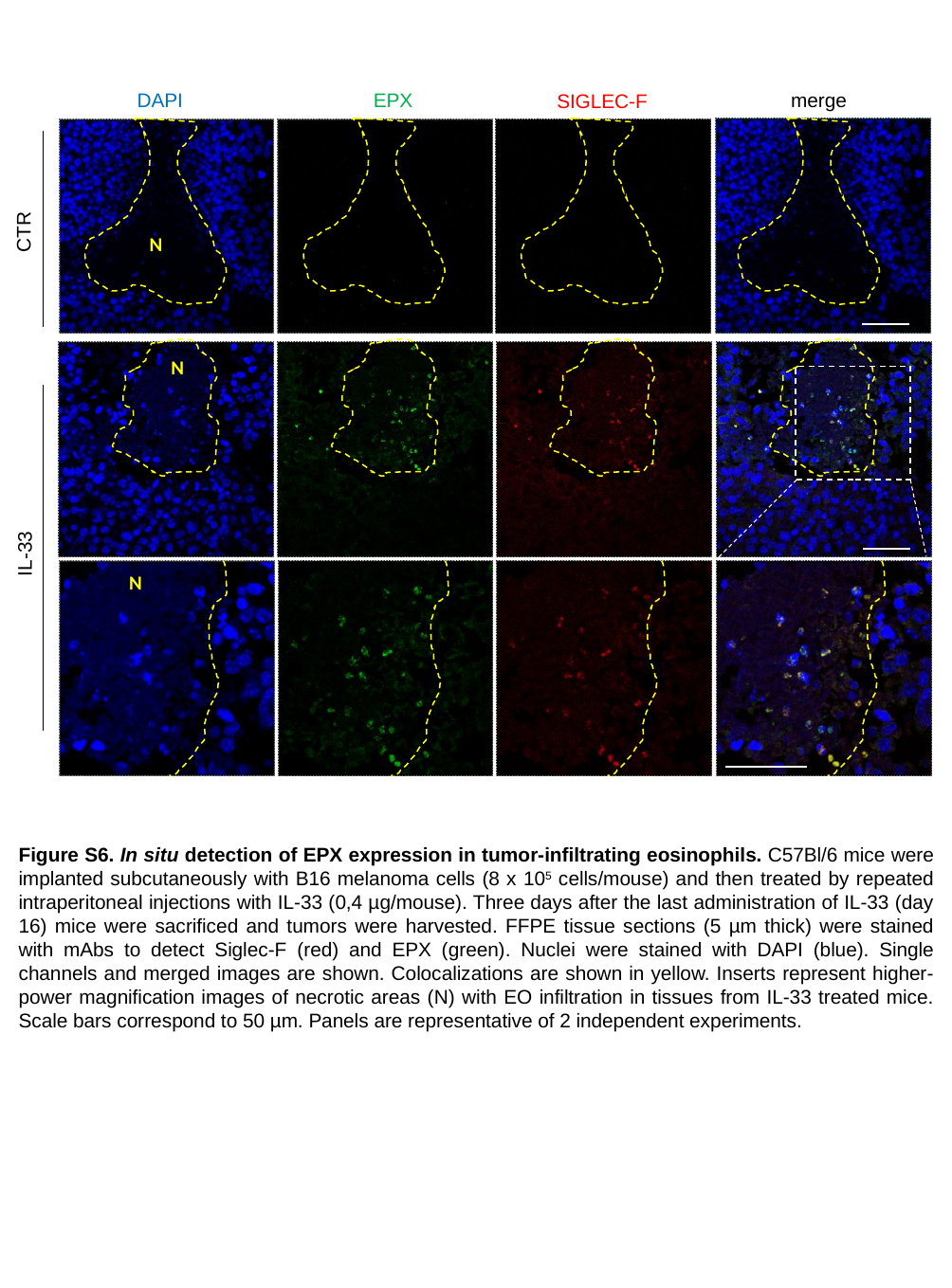

EPX
merge
DAPI
SIGLEC-F
CTR
N
N
IL-33
N
Figure S6. In situ detection of EPX expression in tumor-infiltrating eosinophils. C57Bl/6 mice were implanted subcutaneously with B16 melanoma cells (8 x 10^5^ cells/mouse) and then treated by repeated intraperitoneal injections with IL-33 (0,4 µg/mouse). Three days after the last administration of IL-33 (day 16) mice were sacrificed and tumors were harvested. FFPE tissue sections (5 µm thick) were stained with mAbs to detect Siglec-F (red) and EPX (green). Nuclei were stained with DAPI (blue). Single channels and merged images are shown. Colocalizations are shown in yellow. Inserts represent higher-power magnification images of necrotic areas (N) with EO infiltration in tissues from IL-33 treated mice. Scale bars correspond to 50 µm. Panels are representative of 2 independent experiments.

## Slide 7
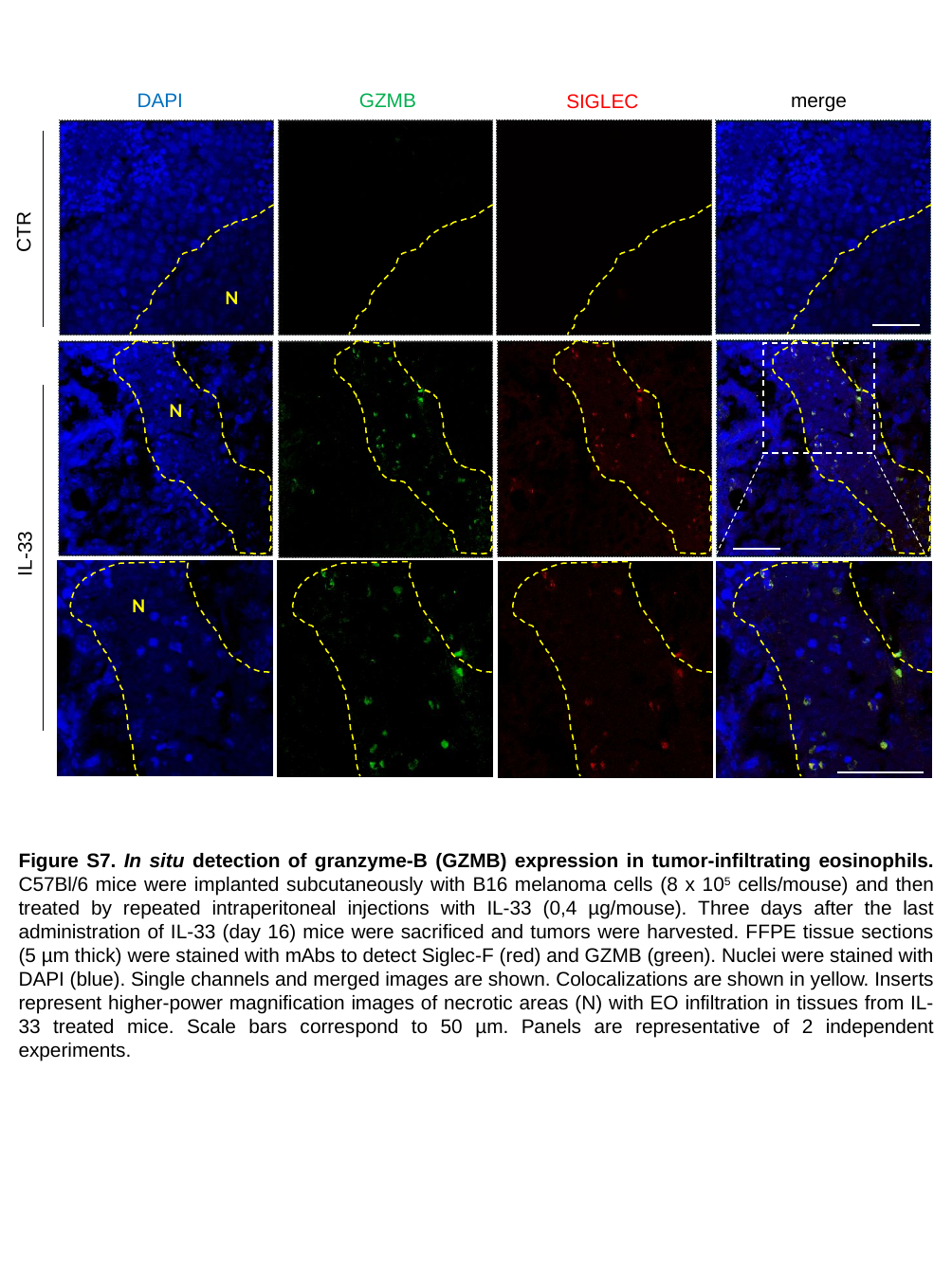

GZMB
merge
DAPI
SIGLEC
CTR
N
N
IL-33
N
Figure S7. In situ detection of granzyme-B (GZMB) expression in tumor-infiltrating eosinophils. C57Bl/6 mice were implanted subcutaneously with B16 melanoma cells (8 x 10^5^ cells/mouse) and then treated by repeated intraperitoneal injections with IL-33 (0,4 µg/mouse). Three days after the last administration of IL-33 (day 16) mice were sacrificed and tumors were harvested. FFPE tissue sections (5 µm thick) were stained with mAbs to detect Siglec-F (red) and GZMB (green). Nuclei were stained with DAPI (blue). Single channels and merged images are shown. Colocalizations are shown in yellow. Inserts represent higher-power magnification images of necrotic areas (N) with EO infiltration in tissues from IL-33 treated mice. Scale bars correspond to 50 µm. Panels are representative of 2 independent experiments.

## Slide 8
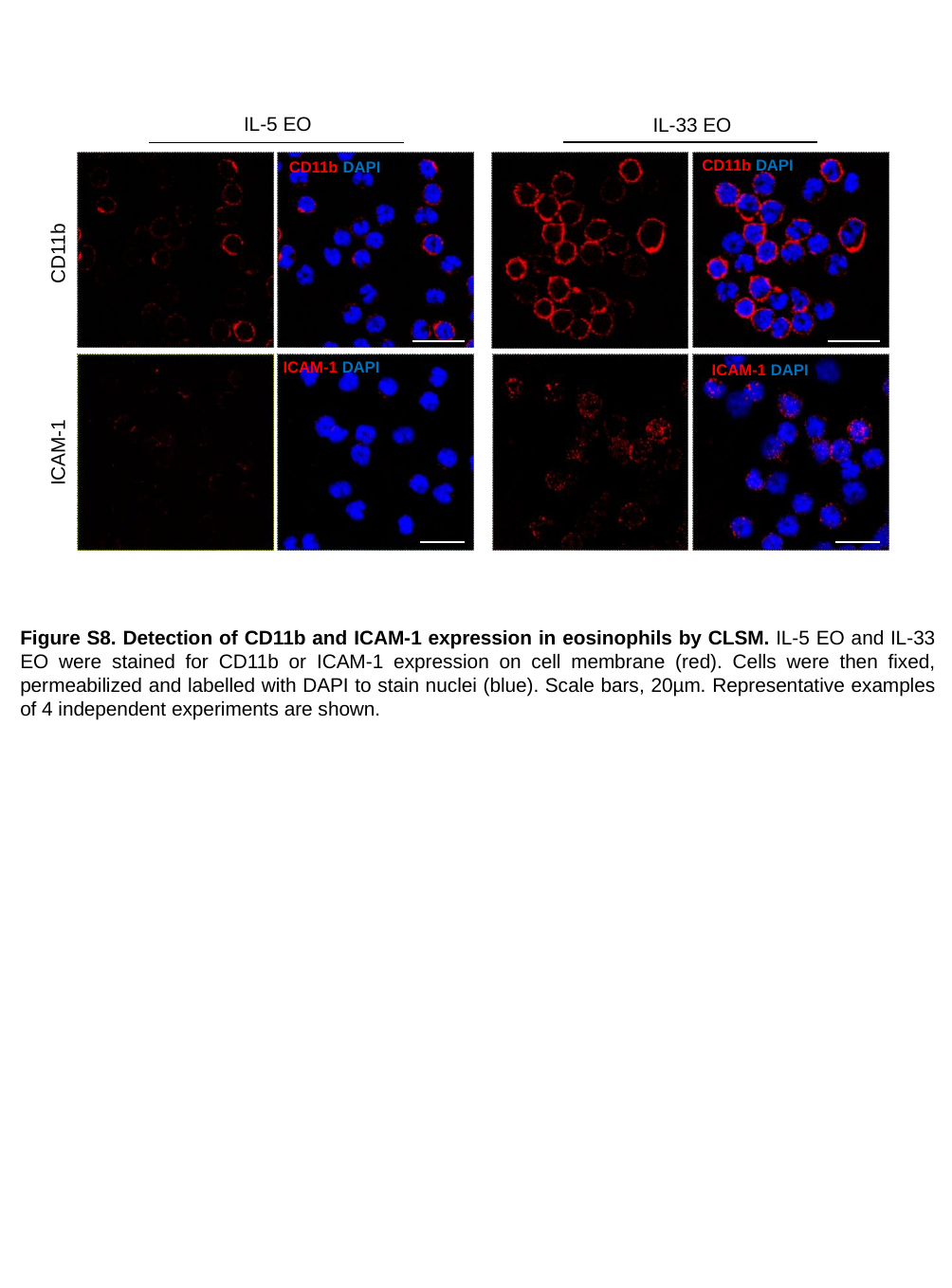

IL-5 EO
IL-33 EO
CD11b DAPI
CD11b DAPI
CD11b
ICAM-1 DAPI
ICAM-1 DAPI
ICAM-1
Figure S8. Detection of CD11b and ICAM-1 expression in eosinophils by CLSM. IL-5 EO and IL-33 EO were stained for CD11b or ICAM-1 expression on cell membrane (red). Cells were then fixed, permeabilized and labelled with DAPI to stain nuclei (blue). Scale bars, 20µm. Representative examples of 4 independent experiments are shown.

## Slide 9
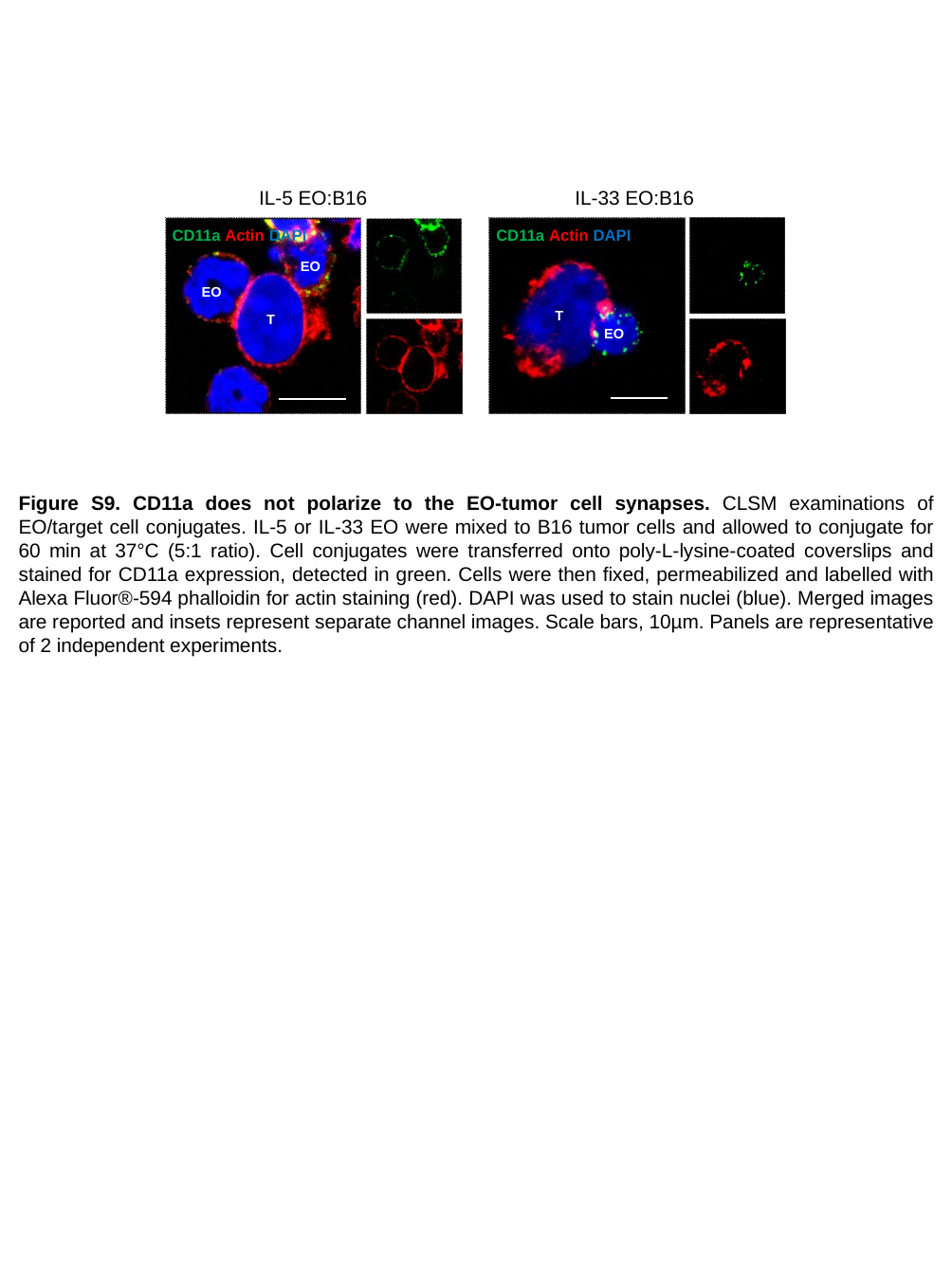

IL-5 EO:B16
IL-33 EO:B16
CD11a/Actin/DAPI
CD11a/Actin/DAPI
EO
EO
T
T
EO
Figure S9. CD11a does not polarize to the EO-tumor cell synapses. CLSM examinations of EO/target cell conjugates. IL-5 or IL-33 EO were mixed to B16 tumor cells and allowed to conjugate for 60 min at 37°C (5:1 ratio). Cell conjugates were transferred onto poly-L-lysine-coated coverslips and stained for CD11a expression, detected in green. Cells were then fixed, permeabilized and labelled with Alexa Fluor®-594 phalloidin for actin staining (red). DAPI was used to stain nuclei (blue). Merged images are reported and insets represent separate channel images. Scale bars, 10µm. Panels are representative of 2 independent experiments.

## Slide 10
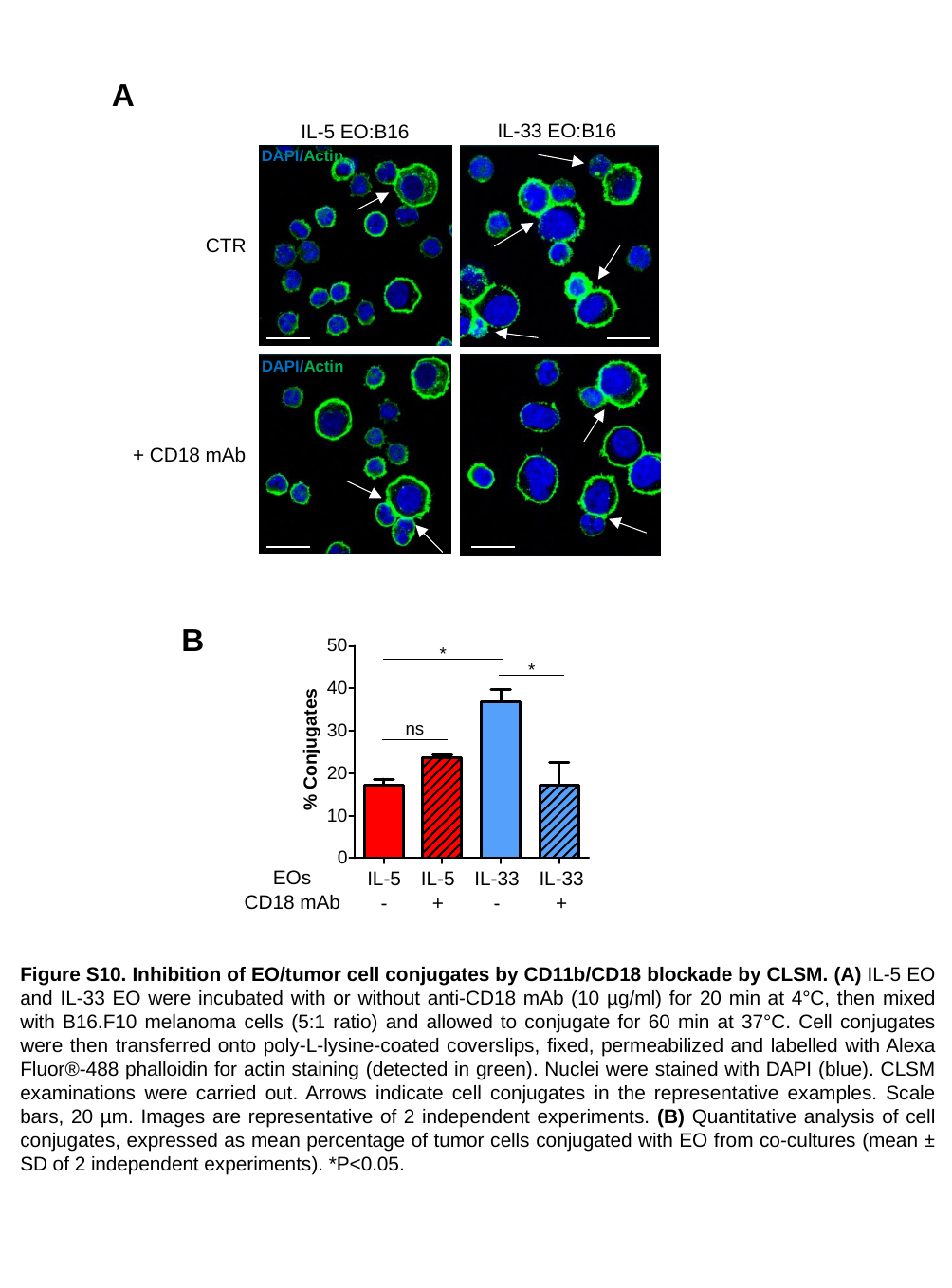

A
IL-33 EO:B16
IL-5 EO:B16
DAPI/Actin
CTR
DAPI/Actin
+ CD18 mAb
B
*
*
ns
| EOs | IL-5 | IL-5 | IL-33 | IL-33 |
| --- | --- | --- | --- | --- |
| CD18 mAb | - | + | - | + |
Figure S10. Inhibition of EO/tumor cell conjugates by CD11b/CD18 blockade by CLSM. (A) IL-5 EO and IL-33 EO were incubated with or without anti-CD18 mAb (10 µg/ml) for 20 min at 4°C, then mixed with B16.F10 melanoma cells (5:1 ratio) and allowed to conjugate for 60 min at 37°C. Cell conjugates were then transferred onto poly-L-lysine-coated coverslips, fixed, permeabilized and labelled with Alexa Fluor®-488 phalloidin for actin staining (detected in green). Nuclei were stained with DAPI (blue). CLSM examinations were carried out. Arrows indicate cell conjugates in the representative examples. Scale bars, 20 µm. Images are representative of 2 independent experiments. (B) Quantitative analysis of cell conjugates, expressed as mean percentage of tumor cells conjugated with EO from co-cultures (mean ± SD of 2 independent experiments). *P<0.05.

## Slide 11
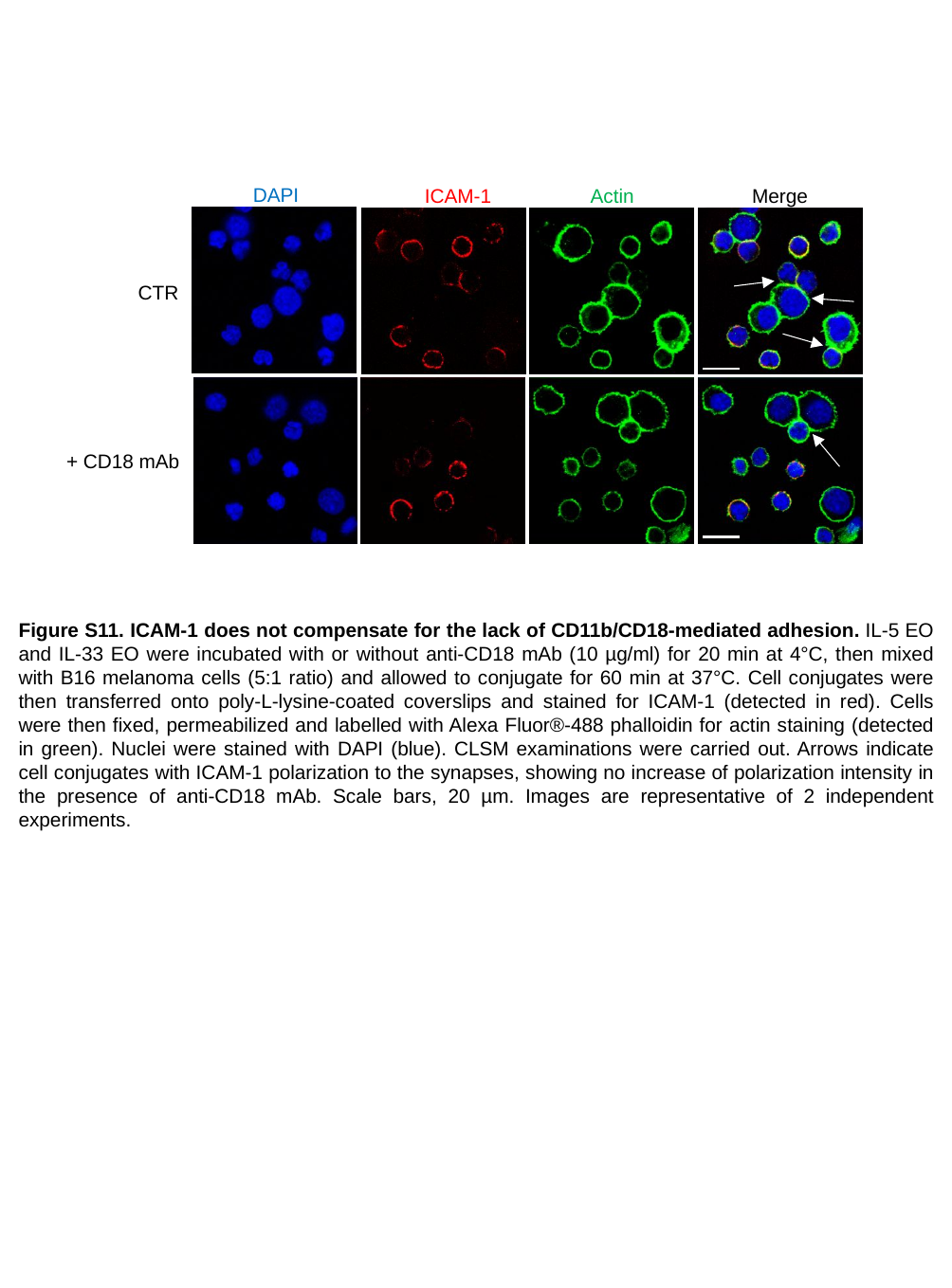

DAPI
ICAM-1
Actin
Merge
CTR
+ CD18 mAb
Figure S11. ICAM-1 does not compensate for the lack of CD11b/CD18-mediated adhesion. IL-5 EO and IL-33 EO were incubated with or without anti-CD18 mAb (10 µg/ml) for 20 min at 4°C, then mixed with B16 melanoma cells (5:1 ratio) and allowed to conjugate for 60 min at 37°C. Cell conjugates were then transferred onto poly-L-lysine-coated coverslips and stained for ICAM-1 (detected in red). Cells were then fixed, permeabilized and labelled with Alexa Fluor®-488 phalloidin for actin staining (detected in green). Nuclei were stained with DAPI (blue). CLSM examinations were carried out. Arrows indicate cell conjugates with ICAM-1 polarization to the synapses, showing no increase of polarization intensity in the presence of anti-CD18 mAb. Scale bars, 20 µm. Images are representative of 2 independent experiments.

## Slide 12
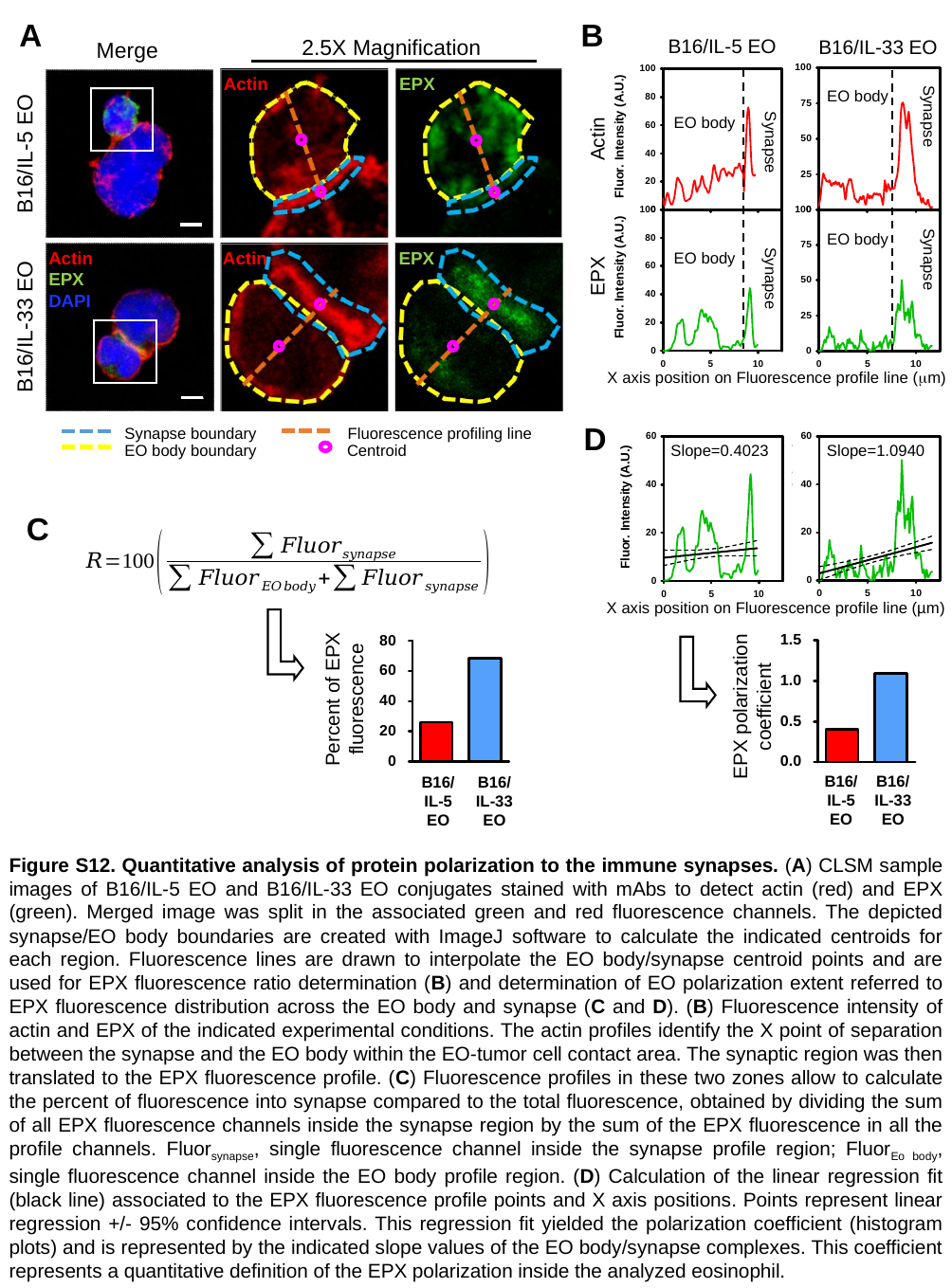

A
B
B16/IL-5 EO
B16/IL-33 EO
EO body
Synapse
EO body
Synapse
Actin
EO body
Synapse
EO body
Synapse
EPX
X axis position on Fluorescence profile line (mm)
2.5X Magnification
Merge
Actin
EPX
Actin
EPX
DAPI
EPX
Actin
B16/IL-5 EO
Actin
EPX
DAPI
Actin
EPX
B16/IL-33 EO
Synapse boundary
Fluorescence profiling line
EO body boundary
Centroid
D
Slope=0.4023
Slope=1.0940
C
X axis position on Fluorescence profile line (µm)
B16/IL-5
EO
B16/IL-33
EO
Percent of EPX fluorescence
EPX polarization coefficient
B16/IL-5
EO
B16/IL-33
EO
Figure S12. Quantitative analysis of protein polarization to the immune synapses. (A) CLSM sample images of B16/IL-5 EO and B16/IL-33 EO conjugates stained with mAbs to detect actin (red) and EPX (green). Merged image was split in the associated green and red fluorescence channels. The depicted synapse/EO body boundaries are created with ImageJ software to calculate the indicated centroids for each region. Fluorescence lines are drawn to interpolate the EO body/synapse centroid points and are used for EPX fluorescence ratio determination (B) and determination of EO polarization extent referred to EPX fluorescence distribution across the EO body and synapse (C and D). (B) Fluorescence intensity of actin and EPX of the indicated experimental conditions. The actin profiles identify the X point of separation between the synapse and the EO body within the EO-tumor cell contact area. The synaptic region was then translated to the EPX fluorescence profile. (C) Fluorescence profiles in these two zones allow to calculate the percent of fluorescence into synapse compared to the total fluorescence, obtained by dividing the sum of all EPX fluorescence channels inside the synapse region by the sum of the EPX fluorescence in all the profile channels. Fluorsynapse, single fluorescence channel inside the synapse profile region; FluorEo body, single fluorescence channel inside the EO body profile region. (D) Calculation of the linear regression fit (black line) associated to the EPX fluorescence profile points and X axis positions. Points represent linear regression +/- 95% confidence intervals. This regression fit yielded the polarization coefficient (histogram plots) and is represented by the indicated slope values of the EO body/synapse complexes. This coefficient represents a quantitative definition of the EPX polarization inside the analyzed eosinophil.
